# Supplementary material for: Internetwork connectivity of molecular networks across species of life
Source: Sci Rep. 2021 Jan 13;11:1168. doi: 10.1038/s41598-020-80745-9 (PMC7806680; doi:10.1038/s41598-020-80745-9)
Supplement: Supplementary file 2 — Supplementary Information 2. [file 41598_2020_80745_MOESM2_ESM.pdf]

Internetwork connectivity of molecular networks across species of life

Tarun Mahajan[1,*], Roy D. Dar[1,2,3,4,*]

1 Department of Bioengineering, University of Illinois at Urbana-Champaign, Urbana, IL, 61801, USA

2 Department of Electrical and Computer Engineering, University of Illinois at Urbana-Champaign, Urbana, IL, 61801, USA

3 Center for Biophysics and Quantitative Biology, University of Illinois at Urbana-Champaign, Urbana, IL, 61801, USA

4 Carl R. Woese Institute for Genomic Biology, University of Illinois at Urbana-Champaign, Urbana, IL, 61801, USA

* Corresponding author. E-mail: tarunm3@illinois.edu
* Corresponding author. E-mail: roydar@illinois.edu

Supplementary Information: Internetwork connectivity of molecular networks across species of life

Table of Contents

Supplementary Table S1: Transcriptional regulatory and protein-protein interaction networks 5

Supplementary methods 6

Simulating multiplex degree-degree coupling and redundancy 6

Multiplex null model 7

Sampling a subset of multiplex with specified degree-degree coupling and redundancy 8

Sampling a random subset of gene-protein pairs from a multiplex with specified degree distributions and redundancy. 9

Supplementary Figure S1: Degree-degree coupling across species for HINT. 11

Supplementary Figure S2: Redundancy coupling across species for HINT. 12

Supplementary Figure S3: Multiplex attack curves for different species with fragmented multiplexes. ... 13

Supplementary Figure S4: Robustness, degree-degree coupling and redundancy coupling versus number of genes in the multiplex. 14

Supplementary Figure S5: 95% CI for robustness versus degree-degree and redundancy couplings plotted in Figure 4B (main text). 15

Supplementary Figure S6: Robustness vs degree-degree and redundancy couplings with different relationship between in-degree and out-degree based degree-degree couplings. 16

Supplementary Figure S7: Redundancy and robustness versus number of pathogen-related genes in the human multiplex against *RanDP* null model. 17

Supplementary Figure S8: Redundancy and robustness versus number of pathogen-related genes in the human multiplex against *RanDP-RZ* null model. 18

Supplementary Figure S9: Redundancy and robustness versus number of pathogen-related genes in the human multiplex against *RanDP-noC$_D$* null model. 19

Supplementary Figure S10: Redundancy and robustness versus number of disease-related genes in the human multiplex against *RanDP* null model. 20

Supplementary Figure S11: Redundancy and robustness versus number of disease-related genes in the human multiplex against *RanDP-RZ* null model. 21

Supplementary Figure S12: Redundancy and robustness versus number of disease-related genes in the human multiplex against *RanDP-noC$_D$* null model. 22

Supplementary Figure S13: Disease- and pathogen-related genes are enriched in the human multiplex... 23

Supplementary Figure S14: Relationship between degree-degree and redundancy couplings across species. 23

Supplementary Figure S15: Simulations showing dependence of robustness on redundancy coupling..... 24
Supplementary Figure S16: Impact of size of multiplex on robustness.. 25
Supplementary Figure S17: Comparison between multiplexes of different sizes can reveal the impact of degree-degree coupling on robustness. 26
Supplementary Figure S18: Comparison between multiplexes of different sizes can reveal the impact of redundancy coupling on robustness. 27
Supplementary Figure S19: Comparing species degree-degree coupling against randomly shuffled multiplexes for BioGRID PPI. 28
Supplementary Figure S20: Comparing species degree-degree coupling against randomly shuffled multiplexes for HINT PPI. 29
Supplementary Figure S21: Multiplex attack curves for different species against the *Multiplex-Configuration* null model. 30
Supplementary Figure S22: Degree-degree and redundancy coupling for species against the Multiplex-Configuration null model. 31
Supplementary Figure S23: Functionally important genes and proteins are topologically important in TRN against RanDP null model. 32
Supplementary Figure S24: Functionally important genes and proteins are topologically important in TRN against RanDP-RZ null model. 33
Supplementary Figure S25: Functionally important genes and proteins are topologically important in TRN against RanDP-noCD null model. 34
Supplementary Figure S26: Functionally important genes and proteins are topologically important in PPI against RanDP null model. 35
Supplementary Figure S27: Functionally important genes and proteins are topologically important in PPI against RanDP-RZ null model. 36
Supplementary Figure S28: Functionally important genes and proteins are topologically important in PPI against RanDP-noCD null model. 37
Supplementary Figure S29: Functionally important genes and proteins are topologically more vulnerable in species multiplex than TRN and PPI against *RanDP* null model. 38
Supplementary Figure S30: Functionally important genes and proteins are topologically more vulnerable in species multiplex than TRN and PPI against *RanDP-RZ* null model. 39
Supplementary Figure S31: Functionally important genes and proteins are topologically more vulnerable in species multiplex than TRN and PPI against *RanDP-noC*$_D$ null model. 40
Supplementary Figure S32: Comparison between k_{in} distribution for functionally important genes and *RanDP* null model: Jensen Shannon Divergence (JSD) between k_{in} 41
Supplementary Figure S33: Comparison between k_{out} distribution for functionally important genes and *RanDP* null model: Jensen Shannon Divergence (JSD) between k_{out} 42
Supplementary Figure S34: Comparison between K distribution for functionally important genes and *RanDP* null model: Jensen Shannon Divergence (JSD) between K 43

Supplementary Figure S35: Comparison between k_{in} distribution for functionally important genes and *RanDP-RZ* null model: Jensen Shannon Divergence (JSD) between k_{in} 44

Supplementary Figure S36: Comparison between k_{out} distribution for functionally important genes and *RanDP-RZ* null model: Jensen Shannon Divergence (JSD) between k_{out} 45

Supplementary Figure S37: Comparison between K distribution for functionally important genes and *RanDP-RZ* null model: Jensen Shannon Divergence (JSD) between K 46

Supplementary Figure S38: Comparison between k_{in} distribution for functionally important genes and *RanDP-noC$_D$* null model: Jensen Shannon Divergence (JSD) between k_{in} 47

Supplementary Figure S39: Comparison between k_{out} distribution for functionally important genes and *RanDP-noC* null model: Jensen Shannon Divergence (JSD) between k_{out} 48

Supplementary Figure S40: Comparison between K distribution for functionally important genes and *RanDP-noC* null model: Jensen Shannon Divergence (JSD) between K 49

Supplementary Figure S41: Functionally important genes and proteins are redundant and essential against *RanDP-noC$_D$* null model. 50

Supplementary Figure S42: Functionally important genes and proteins are redundant and essential against *RanDP* null model 52

References 53

Supplementary Table S1: Transcriptional regulatory and protein-protein interaction networks

Species	Number of Genes/Proteins (TRN, PPI1, PPI2)	%Overlapping Proteome Coverage (TRN-PPI1, TRN-PPI2)	Edges (TRN, PPI1, PPI2)	Average. Degree (k_{in}/k_{out}, K1, K2)	Size of LCC (TRN, PPI1, PPI2)	Reference (TRN, PPI1, PPI2)
H. pylori	1590, 789	7.64, NA	718, 1687, NA	2.94, 8.21, NA	436, 731, NA	[1], [2], NA
M. tuberculosis	1624, 2907	19.74, NA	3212, 8007, NA	1.98, 5.51, NA	1604, 2895, NA	[3], [4], NA
E. coli	1817, 1223, 2172	5.12, 22.64	3072, 1961, 3655	1.70, 3.19, 2.78	1695, 987, 1236	[5], [6], [7]
S. cerevisiae	3712, 4256, 5305	55.56, 82.3	9869, 14891, 23202	2.66, 7.0, 8.52	3707, 4133, 5210	[8], [9], [7]
C. elegans	3351, 3099, 4533	1.6, 4.16	26294, 5347, 12234	7.85, 3.45, 5.22	3351, 2860, 4298	[10], [9], [7]
D. melanogaster	12323, 6677, 7505	47.02, 49.14	157395, 19347, 30181	12.77, 5.80, 7.36	12323, 6540, 7378	[11], [9], [7]
A. thaliana	790, 5779, 5646	0.87, 0.56	1431, 14592, 23410	1.81, 5.05, 8.18	725, 5447, 5159	[12], [9], [7]
M. musculus	2456, 2056, 2429	2.08, 2.75	6490, 2595, 3542	2.64, 2.52, 2.59	2403, 1464, 1485	[13], [9], [7]
H. sapiens	2862, 11833, 12856	7.93, 10.33	8403, 48595, 62435	2.94, 8.21, 9.40	2804, 11731, 12117	[13], [9], [7]

Table showing the characteristics of TRN and PPI networks used for the nine different species. For H, pylori and M. tuberculosis, there is one TRN and one PPI each. For all the other species, we have one TRN and two PPI each. The two PPIs are labeled as PPI1 and PPI2 respectively;

protein degrees for these networks are labeled K1 and K2 respectively. We have also given the corresponding references for each network dataset used in this study.

Abbreviations:

TRN: Transcriptional Regulatory Network
PPI: Protein-Protein Interaction
PPI1: PPI network from the BioGRID database [9] or other species-specific publications [2,4,6].
PPP2: PPI network from the HINT database [7].
LCC: Largest Connected Component is the subset of genes/proteins in TRN/PPI network where every gene/protein is reachable from every other gene/protein.

Definitions:
% Overlapping Proteome Coverage: Percentage of the proteome included in the multiplexes consisting of TRN and PPI1 networks or TRN and PPI2 networks, respectively.
Edges: Number of edges in the TRN, PPI1 or PPI2 networks.

Supplementary methods

Simulating multiplex degree-degree coupling and redundancy

Multiplexes with different degree-degree coupling and redundancy values were generated using a simulated annealing sampling approach [14]. This is achieved by randomly shuffling gene labels in the TRN network and the sampling is biased iteratively to match degree-degree coupling and redundancy with the desired values. Protein labels in the PPI network are kept fixed. The steps are described in Algorithm 1 below.

Algorithm 1:

1. Randomly shuffle gene labels in the TRN network, while protein labels in the PPI network are held constant. Calculate absolute difference between the shuffled and observed degree-degree coupling and redundancy. Let us call these differences $\mathrm{diffcor}^{\mathrm{cur}} = |\mathrm{cor}_{\mathrm{k,K}}^{\mathrm{cur}} - \mathrm{cor}_{\mathrm{k,K}}^{\mathrm{des}}|$ for degree-degree coupling and $\mathrm{diffRed}^{\mathrm{cur}} = |\mathrm{Redundancy}_{12}^{\mathrm{cur}} - \mathrm{Redundancy}_{12}^{\mathrm{des}}|$ for redundancy, where $\mathrm{cor}_{\mathrm{k,K}}^{\mathrm{cur}}$ is the correlation between k (degree) in the shuffled TRN network and K (degree) in the PPI network and $\mathrm{cor}_{\mathrm{k,K}}^{\mathrm{des}}$ is the desired correlation between k and K, $\mathrm{Redundancy}_{12}^{\mathrm{cur}}$ is the redundancy in the shuffled multiplex and $\mathrm{Redundancy}_{12}^{\mathrm{des}}$ is the desired Redundancy. Superscript 'cur' represents that these are the current values of the variables. Save the current gene labeling in the TRN network

2. Randomly select a subset of size N (we use N = 10) from the genes in the TRN network, and randomly shuffle their labels. Save this modified labeling of the TRN as a new variable. Calculate the new proposed difference variables $\mathrm{diffcor}^{\mathrm{prop}} = |\mathrm{cor}_{\mathrm{k,K}}^{\mathrm{prop}} - \mathrm{cor}_{\mathrm{k,K}}^{\mathrm{des}}|$ and $\mathrm{diffRed}^{\mathrm{prop}} = |\mathrm{Redundancy}_{12}^{\mathrm{prop}} - \mathrm{Redundancy}_{12}^{\mathrm{des}}|$as the absolute difference between the degree-degree coupling and redundancy in the new proposed multiplex and the desired

values, respectively. Superscript ‘prop’ represents that these are the proposed values of the variables.

3. Calculate the difference $\Delta = \nu(\text{diffcor}^{\text{cur}} - \text{diffcor}^{\text{prop}}) + (\text{diffRed}^{\text{cur}} - \text{diffRed}^{\text{prop}})$, where ν is a scaling factor; we use $\nu = 10$. If $\Delta >= 0$, save the labeling proposed in step 2 as the current labeling for TRN. Otherwise, accept the labeling proposed in step 2 with probability $e^{\Delta/T}$, where $T = T_0 e^{-\lambda L}$ is a temperature variable, T_0 is the initial temperature (we use $T_0 = 1000$), λ is a rate parameter (we use $\lambda = 0.01$) and L is the iteration number for the simulated annealing loop.
4. Repeat steps 2-3 until $\text{diffcor}^{\text{cur}}$ and $\text{diffRed}^{\text{cur}}$ reach below pre-defined thresholds (we use $\text{diffcor}^{\text{cur}} = 0.001$ and $\text{diffRed}^{\text{cur}} = 3/\sigma(\text{Edges}_{12}^{\text{null}})$ as the thresholds).

The node labeling for TRN achieved at the end of the simulated annealing loop has the desired degree-degree coupling and redundancy between the TRN and PPI layers. Since simulated annealing is a stochastic process, we repeat this procedure multiple times.

We can also match in-degree and out-degree couplings using this algorithm. For this purpose, $\text{diffcor}^{\text{cur}}$ and $\text{diffcor}^{\text{prop}}$ can be decomposed into $\text{diffcor}^{\text{cur}} = \text{diffcor}_{\text{in}}^{\text{cur}} + \text{diffcor}_{\text{out}}^{\text{cur}}$ and $\text{diffcor}^{\text{prop}} = \text{diffcor}_{\text{in}}^{\text{prop}} + \text{diffcor}_{\text{out}}^{\text{prop}}$ respectively, where $\text{diffcor}_{\text{in}}^{\text{cur}} = |\text{cor}_{\mathbf{k_{in},K}}^{\text{cur}} - \text{cor}_{\mathbf{k_{in},K}}^{\text{des}}|$, $\text{diffcor}_{\text{out}}^{\text{cur}} = |\text{cor}_{\mathbf{k_{out},K}}^{\text{cur}} - \text{cor}_{\mathbf{k_{out},K}}^{\text{des}}|$, $\text{diffcor}_{\text{in}}^{\text{prop}} = |\text{cor}_{\mathbf{k_{in},K}}^{\text{prop}} - \text{cor}_{\mathbf{k_{in},K}}^{\text{des}}|$ and $\text{diffcor}_{\text{out}}^{\text{prop}} = |\text{cor}_{\mathbf{k_{out},K}}^{\text{prop}} - \text{cor}_{\mathbf{k_{out},K}}^{\text{des}}|$, $\text{cor}_{\mathbf{k_{in},K}}^{\text{cur}}$ ($\text{cor}_{\mathbf{k_{in},K}}^{\text{prop}}$) and $\text{cor}_{\mathbf{k_{out},K}}^{\text{cur}}$ ($\text{cor}_{\mathbf{k_{out},K}}^{\text{prop}}$) are the correlations between k_{in}, k_{out} in the shuffled TRN network and K in the PPI network respectively and, $\text{cor}_{\mathbf{k_{in},K}}^{\text{des}}$ and $\text{cor}_{\mathbf{k_{out},K}}^{\text{des}}$ are the desired correlations between k_{in}, k_{out} and K respectively.

Multiplex null model

We use two null models to assess robustness of species multiplex--*Zero-Coupling-Zero-Redundancy* and *Multiplex-Configuration* models. The former is used to assess the effect of degree-degree coupling and redundancy on robustness. While the latter model helps to assess the impact of individual network (TRN or PPI) structure on multiplex robustness. These models are generated as follows.

Zero-Coupling-Zero-Redundancy

Under this null model, we generate null multiplexes which have no degree-degree coupling and no redundancy. We generate multiplexes from this model by setting $\text{cor}_{\mathbf{k_{in},K}}^{\text{des}} = \text{cor}_{\mathbf{k_{out},K}}^{\text{des}} = \text{Redundancy}_{12}^{\text{des}} = 0$ in Algorithm 1. This procedure is repeated 100 times to generate a distribution of multiplexes in the null model.

Multiplex-Configuration

Under this model, we generate null multiplex using the well-known configuration model prevalent in network research. The one-to-one correspondence between the genes in TRN and proteins in PPI is fixed. Further, the degree distribution in TRN and PPI is also unchanged. However, the edges are randomly shuffled such that the degree distribution is undisturbed. If the edge shuffling is performed for sufficient iterations, we get a randomized network structure.

Sampling a subset of multiplex with specified degree-degree coupling and redundancy.

Subsets of a multiplex with different degree-degree coupling and redundancy values were sampled using a simulated annealing sampling approach [14]. This is achieved by randomly sampling gene-protein pairs and the sampling is biased iteratively to match degree-degree coupling and redundancy with the desired values. The steps are described in Algorithm 2 below.

Algorithm 2:

1. Randomly sample gene-protein pairs from the multiplex. Calculate absolute difference between the sampled and observed degree-degree coupling and redundancy. Let us call these differences $\mathrm{diffcor}^{\mathrm{cur}} = |\mathrm{cor}_{\mathrm{k,K}}^{\mathrm{cur}} - \mathrm{cor}_{\mathrm{k,K}}^{\mathrm{des}}|$ for degree-degree coupling and $\mathrm{diffRed}^{\mathrm{cur}} = |\mathrm{Redundancy}_{12}^{\mathrm{cur}} - \mathrm{Redundancy}_{12}^{\mathrm{des}}|$ for redundancy, where $\mathrm{cor}_{\mathrm{k,K}}^{\mathrm{cur}}$ is the correlation between k (degree) in the sampled TRN network and K (degree) in the sampled PPI network and $\mathrm{cor}_{\mathrm{k,K}}^{\mathrm{des}}$ is the desired correlation between k and K, $\mathrm{Redundancy}_{12}^{\mathrm{cur}}$ is the redundancy in the sampled multiplex and $\mathrm{Redundancy}_{12}^{\mathrm{des}}$ is the desired Redundancy. Superscript 'cur' represents that these are the current values of the variables. Save the current labels of the sampled gene-protein pairs in the multiplex.
2. Randomly select a subset of size N (we use N = 5) from the set of gene-protein pairs sampled in the previous step. Also, randomly select N gene-protein pairs from the set of gene-protein pairs not in the sampled set. Swap the N gene-proteins from the sampled set with the N genes in the not-sampled set. Save this sampled set as a new variable. Calculate the new proposed difference variables $\mathrm{diffcor}^{\mathrm{prop}} = |\mathrm{cor}_{\mathrm{k,K}}^{\mathrm{prop}} - \mathrm{cor}_{\mathrm{k,K}}^{\mathrm{des}}|$ and $\mathrm{diffRed}^{\mathrm{prop}} = |\mathrm{Redundancy}_{12}^{\mathrm{prop}} - \mathrm{Redundancy}_{12}^{\mathrm{des}}|$as the absolute difference between the degree-degree coupling and redundancy in the new proposed sampled subset of the multiplex and the desired values, respectively. Superscript 'prop' represents that these are the proposed values of the variables.
3. Calculate the difference $\Delta = \nu(\mathrm{diffcor}^{\mathrm{cur}} - \mathrm{diffcor}^{\mathrm{prop}}) + (\mathrm{diffRed}^{\mathrm{cur}} - \mathrm{diffRed}^{\mathrm{prop}})$, where ν is a scaling factor; we use $\nu = 10$. If $\Delta >= 0$, save the proposed subset in step 2 as the current subset. Otherwise, accept the proposed subset in step 2 with probability $e^{\Delta/T}$, where $T = T_0 e^{-\lambda L}$ is a temperature variable, T_0 is the initial temperature (we use $T_0 = 1000$), λ is a rate parameter (we use $\lambda = 0.01$) and L is the iteration number for the simulated annealing loop.

4. Repeat steps 2-3 until $\text{diffcor}^{\text{cur}}$ and $\text{diffRed}^{\text{cur}}$ reach below pre-defined thresholds (we use $\text{diffcor}^{\text{cur}} = 0.001$ and $\text{diffRed}^{\text{cur}} = 10$ as the thresholds).

The sampled set achieved at the end of the simulated annealing loop has the desired degree-degree coupling and redundancy between the TRN and PPI layers of the sampled subset of the multiplex. Since simulated annealing is a stochastic process, we repeat this procedure multiple times.

We can also match in-degree and out-degree couplings using this algorithm. For this purpose, $\text{diffcor}^{\text{cur}}$ and $\text{diffcor}^{\text{prop}}$ can be decomposed into $\text{diffcor}^{\text{cur}} = \text{diffcor}^{\text{cur}}_{\text{in}} + \text{diffcor}^{\text{cur}}_{\text{out}}$ and $\text{diffcor}^{\text{prop}} = \text{diffcor}^{\text{prop}}_{\text{in}} + \text{diffcor}^{\text{prop}}_{\text{out}}$ respectively, where $\text{diffcor}^{\text{cur}}_{\text{in}} = |\text{cor}^{\text{cur}}_{\mathbf{k_{in}},\mathbf{K}} - \text{cor}^{\text{des}}_{\mathbf{k_{in}},\mathbf{K}}|$, $\text{diffcor}^{\text{cur}}_{\text{out}} = |\text{cor}^{\text{cur}}_{\mathbf{k_{out}},\mathbf{K}} - \text{cor}^{\text{des}}_{\mathbf{k_{out}},\mathbf{K}}|$, $\text{diffcor}^{\text{prop}}_{\text{in}} = |\text{cor}^{\text{prop}}_{\mathbf{k_{in}},\mathbf{K}} - \text{cor}^{\text{des}}_{\mathbf{k_{in}},\mathbf{K}}|$ and $\text{diffcor}^{\text{prop}}_{\text{out}} = |\text{cor}^{\text{prop}}_{\mathbf{k_{out}},\mathbf{K}} - \text{cor}^{\text{des}}_{\mathbf{k_{out}},\mathbf{K}}|$, $\text{cor}^{\text{cur}}_{\mathbf{k_{in}},\mathbf{K}}$ ($\text{cor}^{\text{prop}}_{\mathbf{k_{in}},\mathbf{K}}$) and $\text{cor}^{\text{cur}}_{\mathbf{k_{out}},\mathbf{K}}$ ($\text{cor}^{\text{prop}}_{\mathbf{k_{out}},\mathbf{K}}$) are the correlations between k_{in}, k_{out} in the sampled TRN network and K in the sampled PPI network respectively and, $\text{cor}^{\text{des}}_{\mathbf{k_{in}},\mathbf{K}}$ and $\text{cor}^{\text{des}}_{\mathbf{k_{out}},\mathbf{K}}$ are the desired correlations between k_{in}, k_{out} and K respectively.

Sampling a random subset of gene-protein pairs from a multiplex with specified degree distributions and redundancy.

Matching gene and protein degrees to a functional set of gene-protein pairs

Subsets of a multiplex were sampled using a simulated annealing sampling approach [14]. This is achieved by randomly sampling gene-protein pairs and the sampling is biased iteratively to match gene and protein degrees to a functional set of gene-protein pairs. Further, redundancy can also be matched to a desired value. This method was used to generate the *RanDP* and *RanDP-RZ* null models. Steps for the method are described in Algorithm 3 below.

Algorithm 3:

1. Randomly sample gene-protein pairs from the multiplex. Calculate absolute difference between the sampled and desired network degrees (TRN and PPI) and redundancy. Let us call these differences $\text{diffkin}^{\text{cur}} = |\text{k}^{\text{cur}}_{\text{in}} - \text{k}^{\text{func}}_{\text{in}}|$, $\text{diffkout}^{\text{cur}} = |\text{k}^{\text{cur}}_{\text{out}} - \text{k}^{\text{func}}_{\text{out}}|$, $\text{diffK}^{\text{cur}} = |\text{K}^{\text{cur}} - \text{K}^{\text{func}}|$ and $\text{diffRed}^{\text{cur}} = |\text{Redundancy}^{\text{cur}}_{12} - \text{Redundancy}^{\text{des}}_{12}|$ for TRN in-degrees, TRN out-degrees, PPI degrees and redundancy, respectively. Here, $\text{k}^{\text{cur}}_{\text{in}}$, $\text{k}^{\text{cur}}_{\text{out}}$, K^{cur}, $\text{k}^{\text{func}}_{\text{in}}$, $\text{k}^{\text{func}}_{\text{out}}$ and K^{func} are the vectors of TRN in-degrees in the sampled subset, TRN out-degrees in the sampled subset, PPI degrees in the sampled subset, TRN in-degrees in the functional subset, TRN out-degrees in the functional subset and PPI degrees in the functional subset, respectively. Further, $\text{Redundancy}^{\text{cur}}_{12}$ is the redundancy in the sampled subset and $\text{Redundancy}^{\text{des}}_{12}$ is the desired Redundancy. Superscript 'cur' represents that these are the current values of the variables. Save the current labels of the sampled gene-protein pairs in the multiplex.

2. Randomly select a subset of size N (we use N = 5) from the set of gene-proteins sampled in the previous step. Also, randomly select N gene-protein pairs from the set of gene-

protein pairs not in the sampled set. Swap the N gene-proteins from the sampled set with the N genes in the not-sampled set. Save this sampled set as a new variable. Calculate the new proposed difference variables, $\mathrm{diffkin^{prop}} = |k_{in}^{prop} - k_{in}^{func}|$, $\mathrm{diffkout^{prop}} = |k_{out}^{prop} - k_{out}^{func}|$, $\mathrm{diffK^{prop}} = |K^{prop} - K^{func}|$ and $\mathrm{diffRed^{prop}} = |\mathrm{Redundancy}_{12}^{prop} - \mathrm{Redundancy}_{12}^{des}|$ as the absolute difference between the sampled and desired network degrees (TRN and PPI) and redundancy in the new proposed sampled subset of the multiplex. Superscript 'prop' represents that these are the proposed values of the variables.

3. Calculate the difference $\Delta = (\mathrm{diffkin^{cur}} - \mathrm{diffkin^{prop}}) + \nu_1(\mathrm{diffkout^{cur}} - \mathrm{diffkout^{prop}}) + \nu_2(\mathrm{diffK^{cur}} - \mathrm{diffK^{prop}}) + \nu_3(\mathrm{diffRed^{cur}} - \mathrm{diffRed^{prop}})$, where ν_1, ν_2 and ν_3 are scaling factors; we use $\nu_1 = 2$, $\nu_2 = 1$ and $\nu_3 = 1$. If $\Delta >= 0$, save the proposed subset in step 2 as the current subset. Otherwise, accept the proposed subset in step 2 with probability $e^{\Delta/T}$, where $T = T_0 e^{-\lambda L}$ is a temperature variable, T_0 is the initial temperature (we use $T_0 = 1000$), λ is a rate parameter (we use $\lambda = 0.01$) and L is the iteration number for the simulated annealing loop.

4. Repeat steps 2-3 for a predetermined number of iterations; we use 10^6 iterations.

The sampled set achieved at the end of the simulated annealing loop has the desired network degrees and redundancy between the TRN and PPI layers of the sampled subset of the multiplex. Since simulated annealing is a stochastic process, we repeat this procedure multiple times.

An alternative method to sample subsets that match sampled network degrees to a functional subset is to bin the degrees in a 3D space. However, this becomes problematic as the number of gene-protein pairs in each bin drastically reduces as we move to a 3D space. For Algorithm 3, we bin k_{in}, k_{out} and K separately into 1D bins and then sample subsets.

Matching gene and protein degree distributions to a functional set of gene-protein pairs

Algorithm 3 is restrictive in the sense that it ensures that the degree tuple (k_{in}, k_{out}, K) in the sampled subset matches a functional set of gene-protein pairs. This ensures that the sampled subset matches the functional set in C_D. For the *RanDP-noC$_D$* null model, we relax this restriction. To achieve this, we replace degree vectors in Algorithm 3 with degree distributions. Further, *RanDP-noC$_D$* does not impose a redundancy condition on the sampled subsets. Therefore, redundancy-related conditions are omitted from Algorithm 3 for *RanDP-noC$_D$*. With these modifications, Algorithm 3 samples subsets which match only in degree distributions to the functional set.

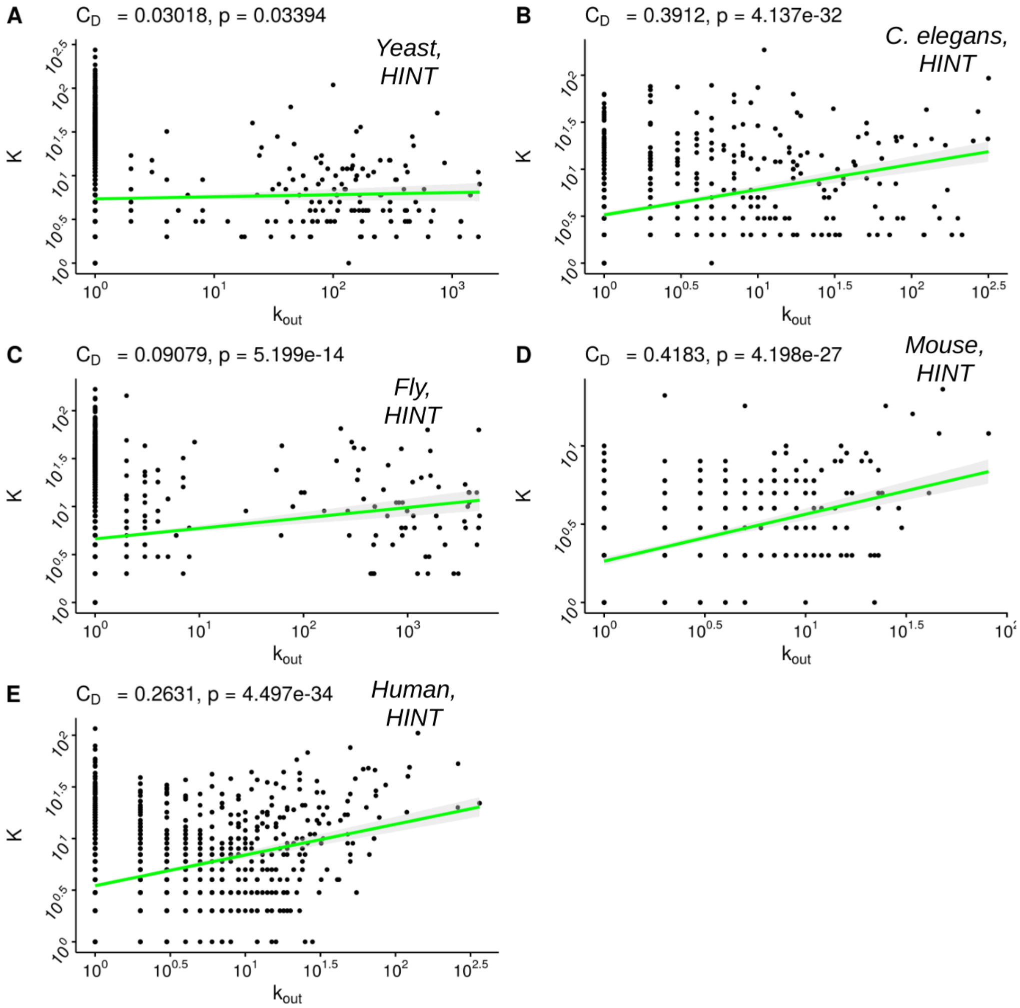

Supplementary Figure S1: Degree-degree coupling across species for HINT. Scatter plot for degree (K) of proteins in the PPI network versus out-degree (k_{out}) of genes in TRN for eukaryotes, A) yeast, B) *C. elegans*, C) fly, D) mouse and E) human. Both K and k_{out} values have been log-transformed after adding 1. The PPI networks are for the HINT database (Methods). Linear interpolated fits between K and k_{out} are also shown (green line) with 95% confidence region shaded in gray. Degree-degree coupling (C_D) values and corresponding p-values (two-tailed z-test using Fisher's z-transformation) are also shown.

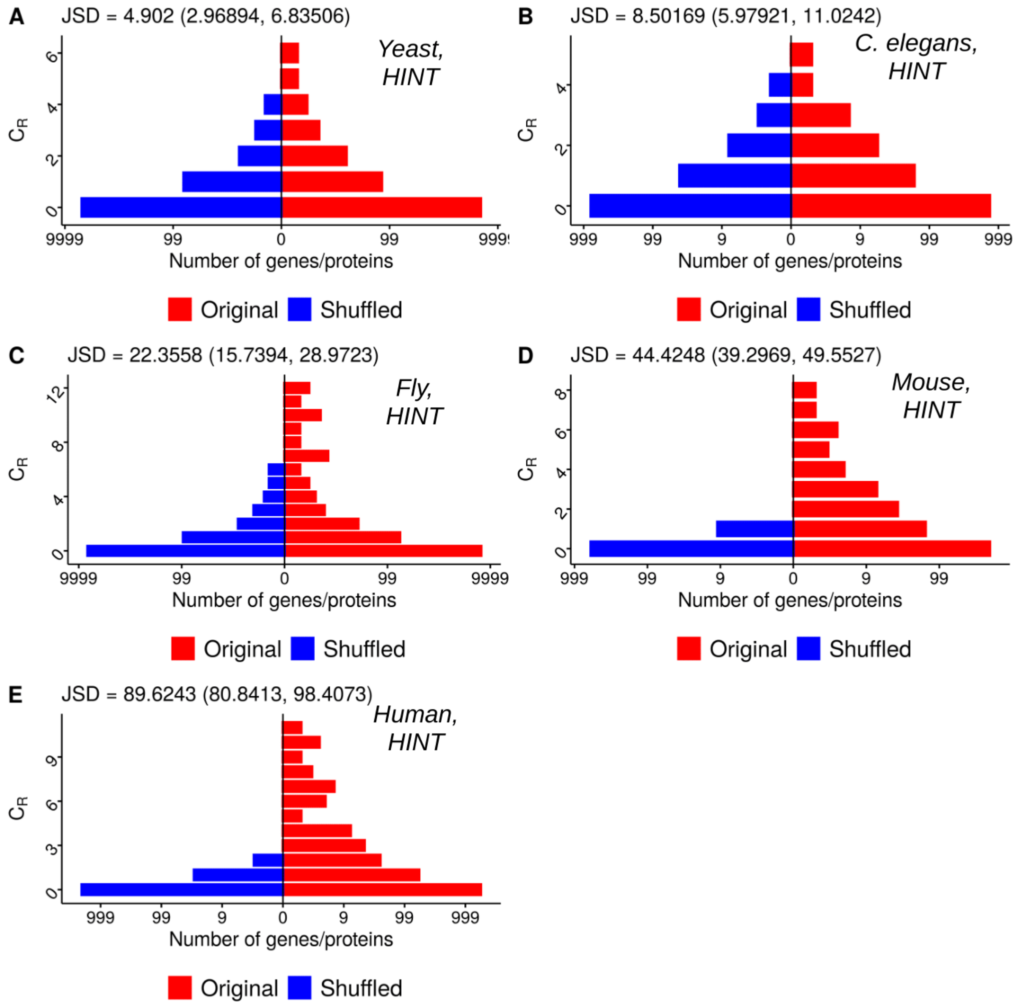

Supplementary Figure S2: Redundancy coupling across species for HINT. Distribution of redundancy (CR) for gene-protein pairs for species multiplex and the randomly shuffled null model for eukaryotes, A) yeast, B) C. elegans, C) fly, D) mouse and E) human. For each gene-protein pair, C_R is quantified by the number of redundant edges incident on that gene-protein pair. Shuffled null model is generated by randomly shuffling labels on genes in TRN, while keeping protein labels fixed in PPI. Jensen Shannon divergence (JSD) (along with 95% CI) between distributions of C_R in organismal and shuffled multiplexes is also shown.

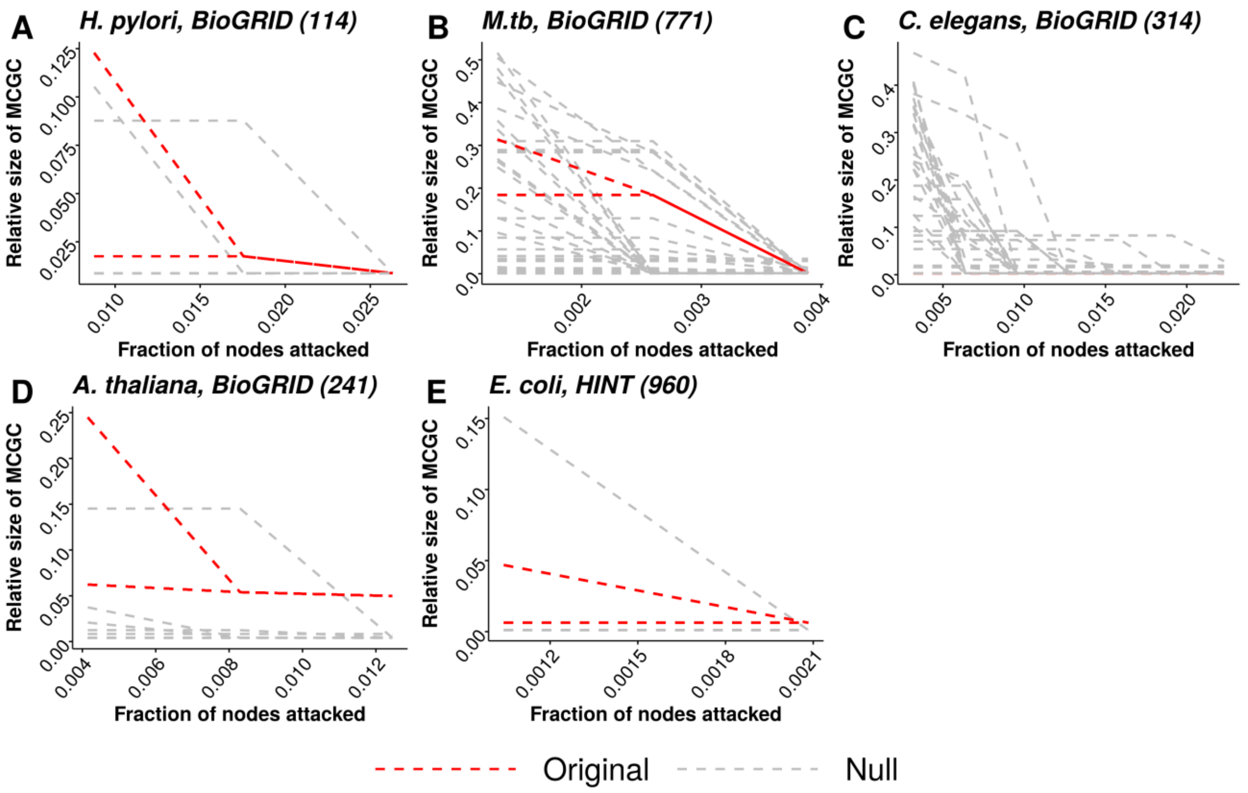

Supplementary Figure S3: Multiplex attack curves for different species with fragmented multiplexes. Relative size of Mutually Connected Giant Component (MCGC) is plotted as a function of the fraction of gene-protein pairs attacked and removed from the multiplex (Methods). Attack curves are shown for six species multiplexes which were not considered in our final analysis. PPI database for a given panel is annotated next to the species name. Along with the attack curves for the species (red), attack curves for the *Zero-Coupling-Zero-Redundancy* null model are also shown (gray). Under this null model, multiplexes have no degree-degree coupling and no redundancy.

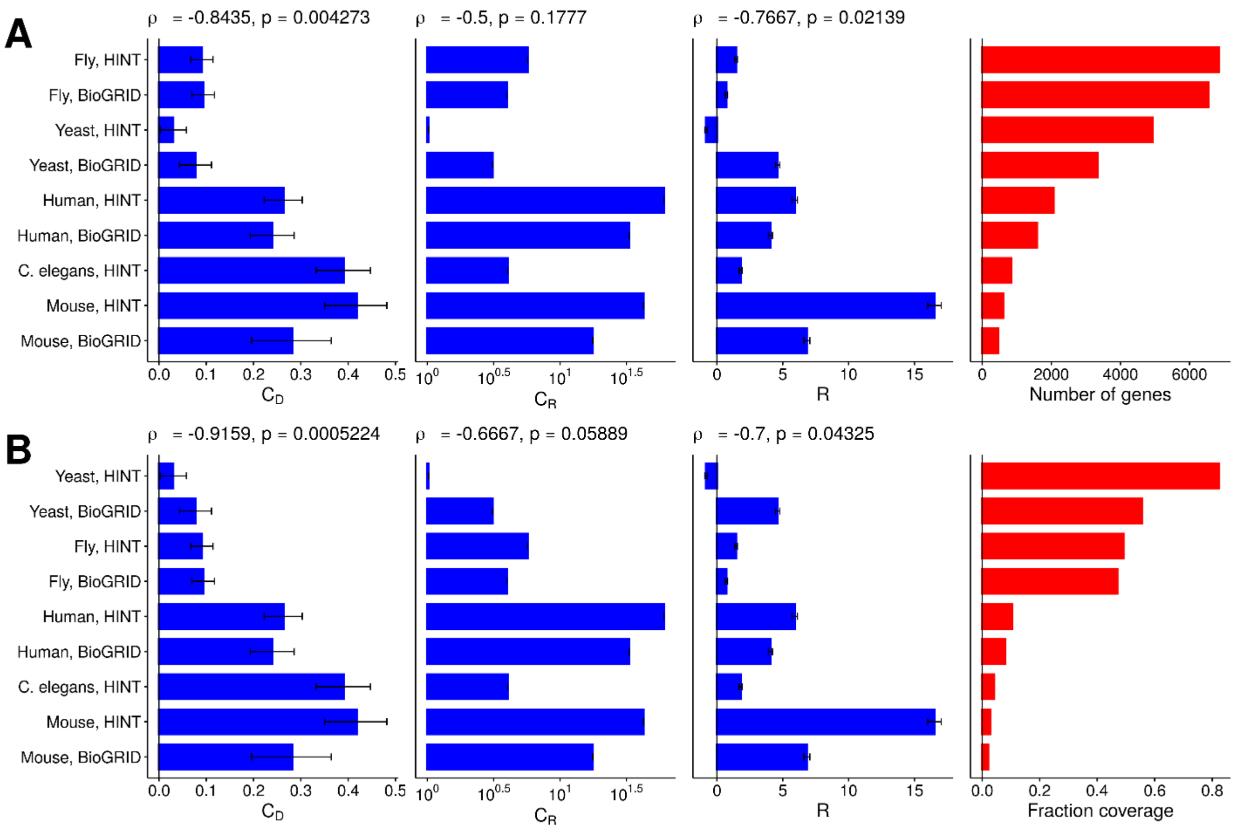

Supplementary Figure S4: Robustness, degree-degree coupling and redundancy coupling versus number of genes in the multiplex. A) Degree-degree coupling (C_D), redundancy coupling (C_R) and robustness (R) versus number of genes in the multiplex. Spearman's correlation coefficient (with p-values) with number of genes is also mentioned. B) Degree-degree coupling (C_D), redundancy coupling (C_R) and robustness (R) versus number of fraction coverage (number of genes in the multiplex divided by the total number of protein-coding genes). Spearman's correlation coefficient (with p-values) with number of genes is also mentioned. In all the panels, error bars show 95% CI.

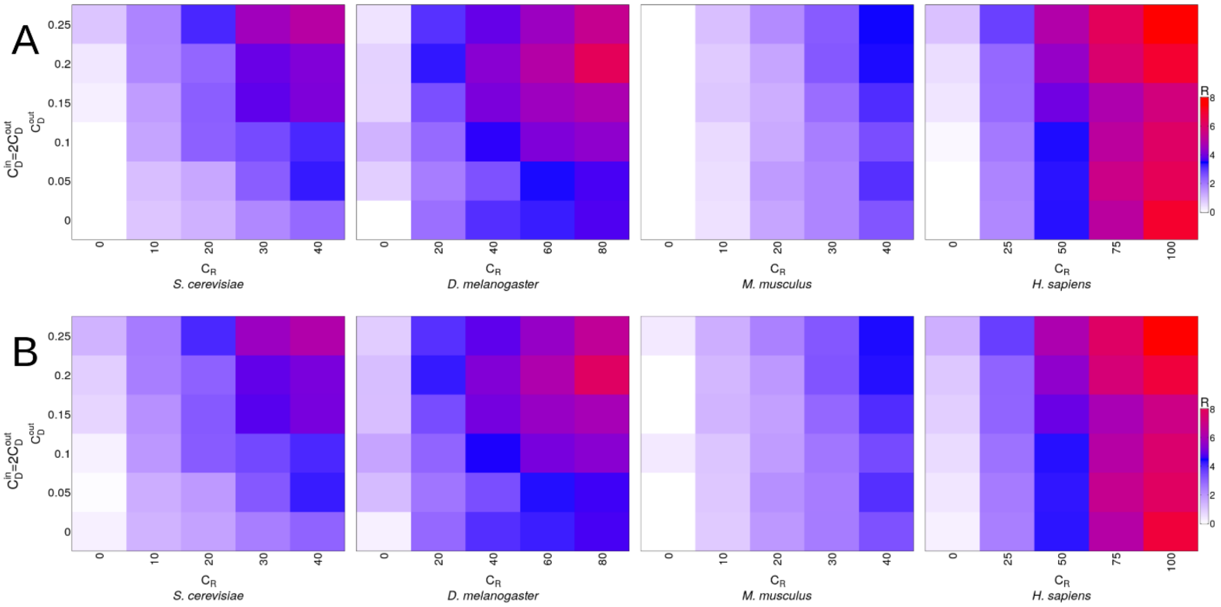

Supplementary Figure S5: 95% CI for robustness versus degree-degree and redundancy couplings plotted in Figure 4B (main text). A) Lower interval for the 95% CI for Figure 4B. Upper interval for the 95% CI for Figure 4B.

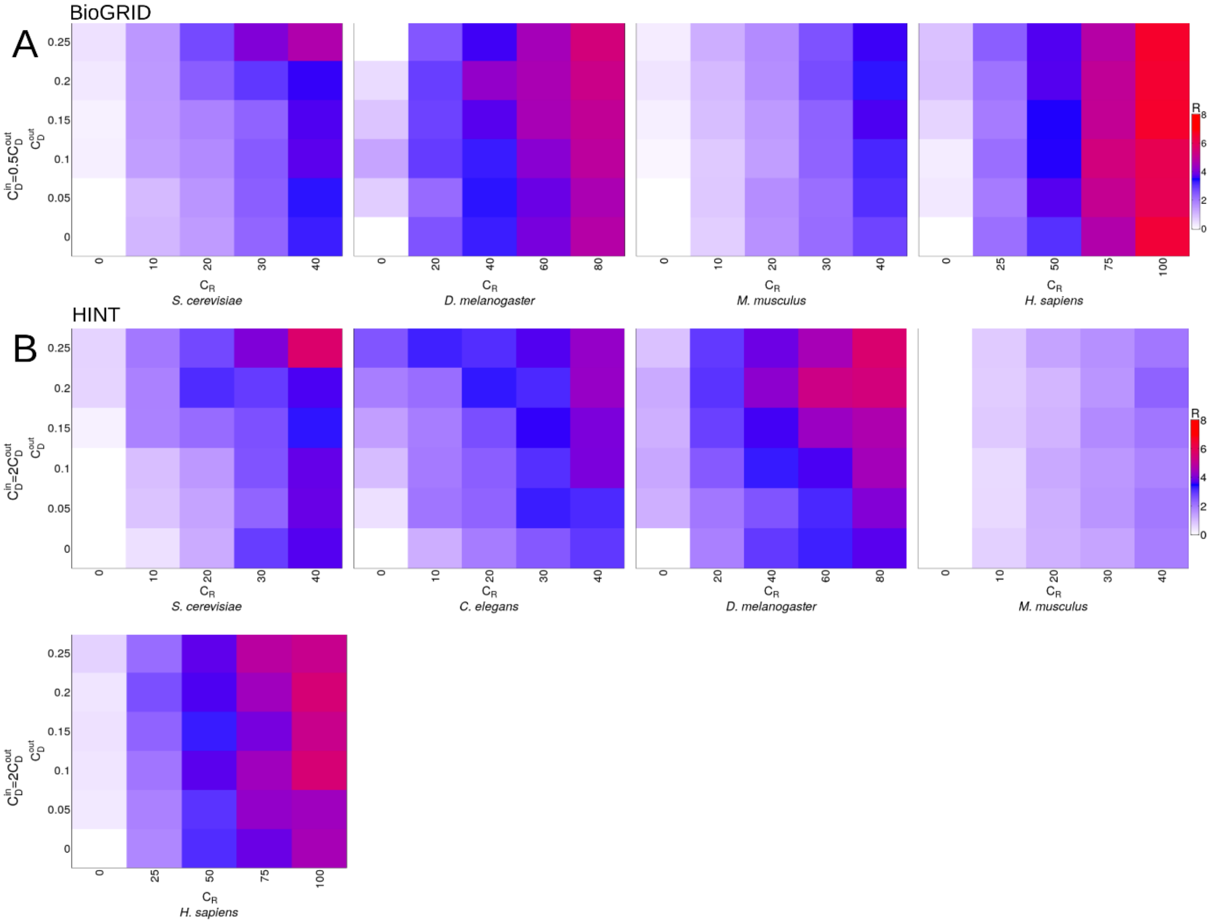

Supplementary Figure S6: Robustness vs degree-degree and redundancy couplings with different relationship between in-degree and out-degree based degree-degree couplings. We sample a subset of gene-protein pairs from the species multiplexes (sizes for the subsets are: *S. cerevisiae*-1000, *C. elegans*-500, *D. melanogaster*-2000, *M. musculus*-300, *H. sapiens*-500) with specific C_D and C_R values. We repeat the sampling 100 times. We explore C_D and C_R over a grid. For each point over the 2D grid, the heatmap shows the robustness (R) value. R is computed by comparing *RobustArea* of any point over the grid against the lower-left point of the grid (with $C_D = 0$, $C_R = 0$). For each point over the grid, for each of the sampled subset, targeted attack is performed. Mean R values are shown here. A) $C_D^{in} = 0.5\ C_D^{out}$. BioGRID PPI networks are used. A) $C_D^{in} = 2\ C_D^{out}$. HINT PPI networks are used. C_D^{out}: degree-degree coupling between k_{out} and K, C_D^{in}: degree-degree coupling between k_{in} and K.

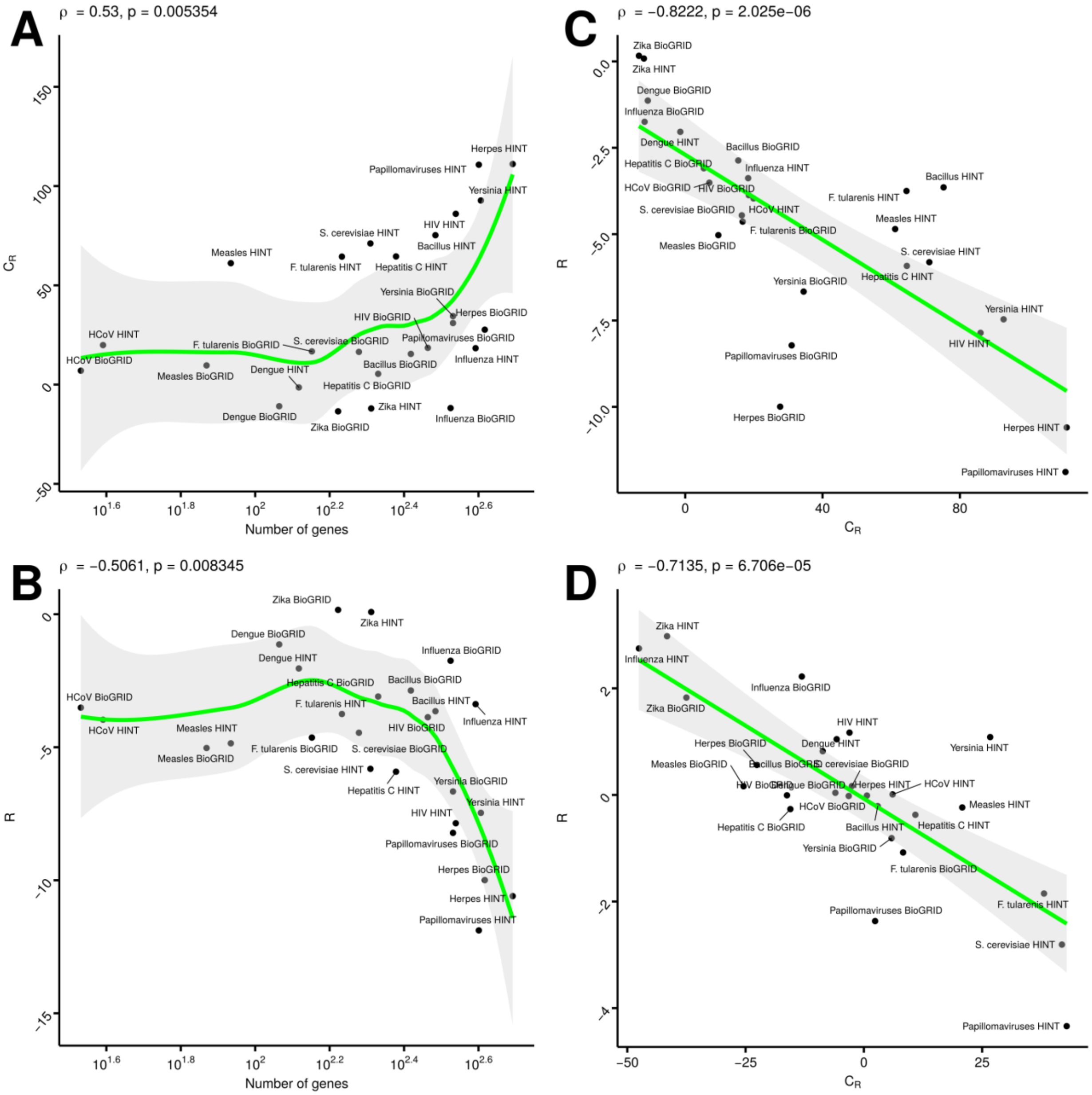

Supplementary Figure S7: Redundancy and robustness versus number of pathogen-related genes in the human multiplex against *RanDP* null model. A) C_R for pathogen-related genes (against *RanDP* null model) versus number of pathogen genes. B) R for pathogen-related genes (against *RanDP* null model) versus number of pathogen genes. C) R versus C_R for pathogen-related genes against *RanDP* null model. D) R versus C_R for pathogen-related genes while controlling for number of genes. R and C_R are regressed against number of genes using loess regression and residuals are plotted. Pathogen names are annotated with the PPI database used for the analysis. For all the panels, Spearman rank correlation values, ρ, are also listed along with p-values (two-tailed z-test using Fisher's z-transformation) are also shown.

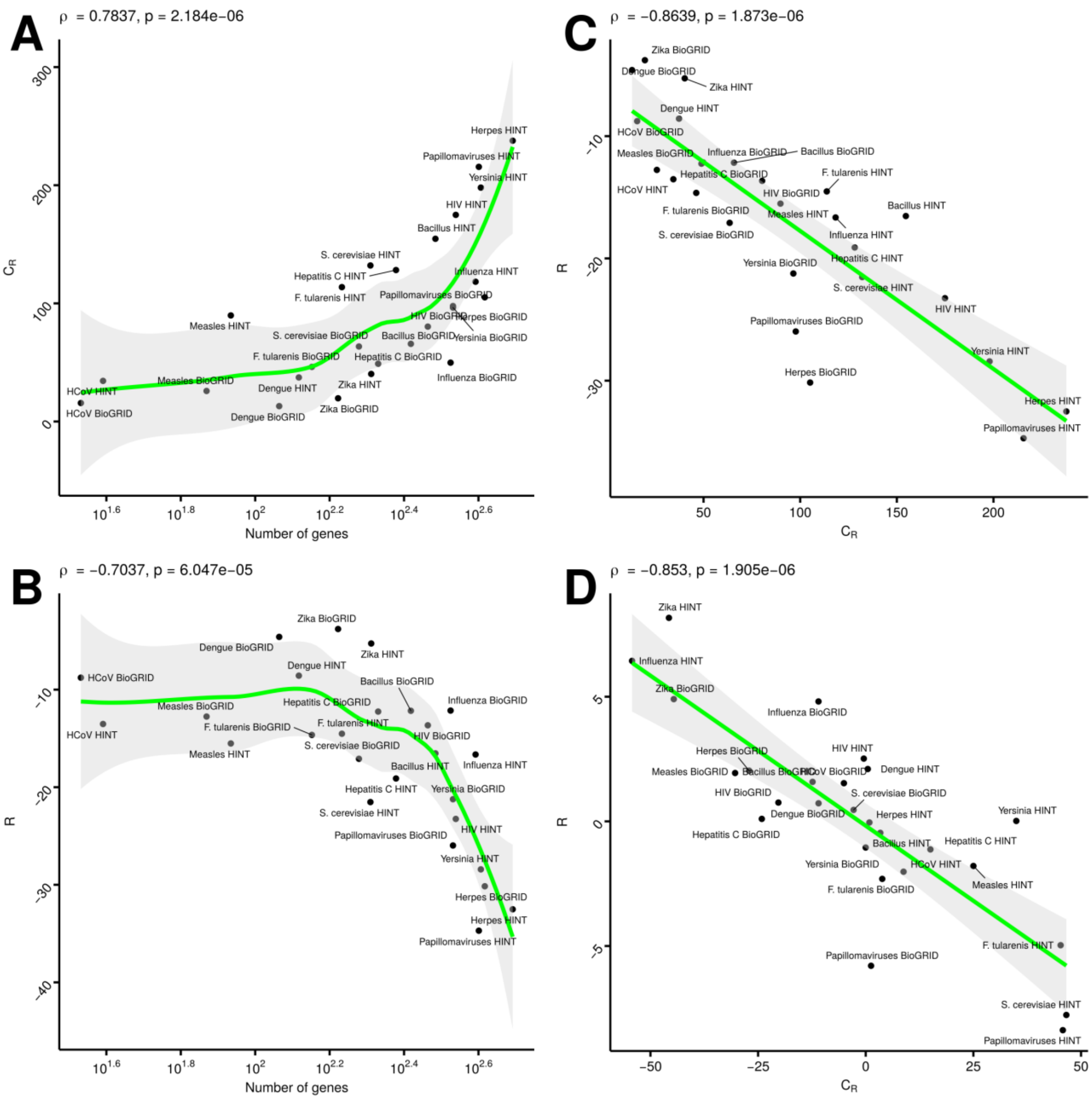

Supplementary Figure S8: Redundancy and robustness versus number of pathogen-related genes in the human multiplex against *RanDP-RZ* null model. A) C_R for pathogen-related genes (against *RanDP-RZ* null model) versus number of pathogen genes. B) R for pathogen-related genes (against *RanDP-RZ* null model) versus number of pathogen genes. C) R versus C_R for pathogen-related genes against *RanDP-RZ* null model. D) R versus C_R for pathogen-related genes while controlling for number of genes. R and C_R are regressed using loess regression against number of genes and residuals are plotted. Pathogen names are annotated with the PPI database used for the analysis. For all the panels, Spearman rank correlation values, ρ, are also listed along with p-values (two-tailed z-test using Fisher's z-transformation) are also shown.

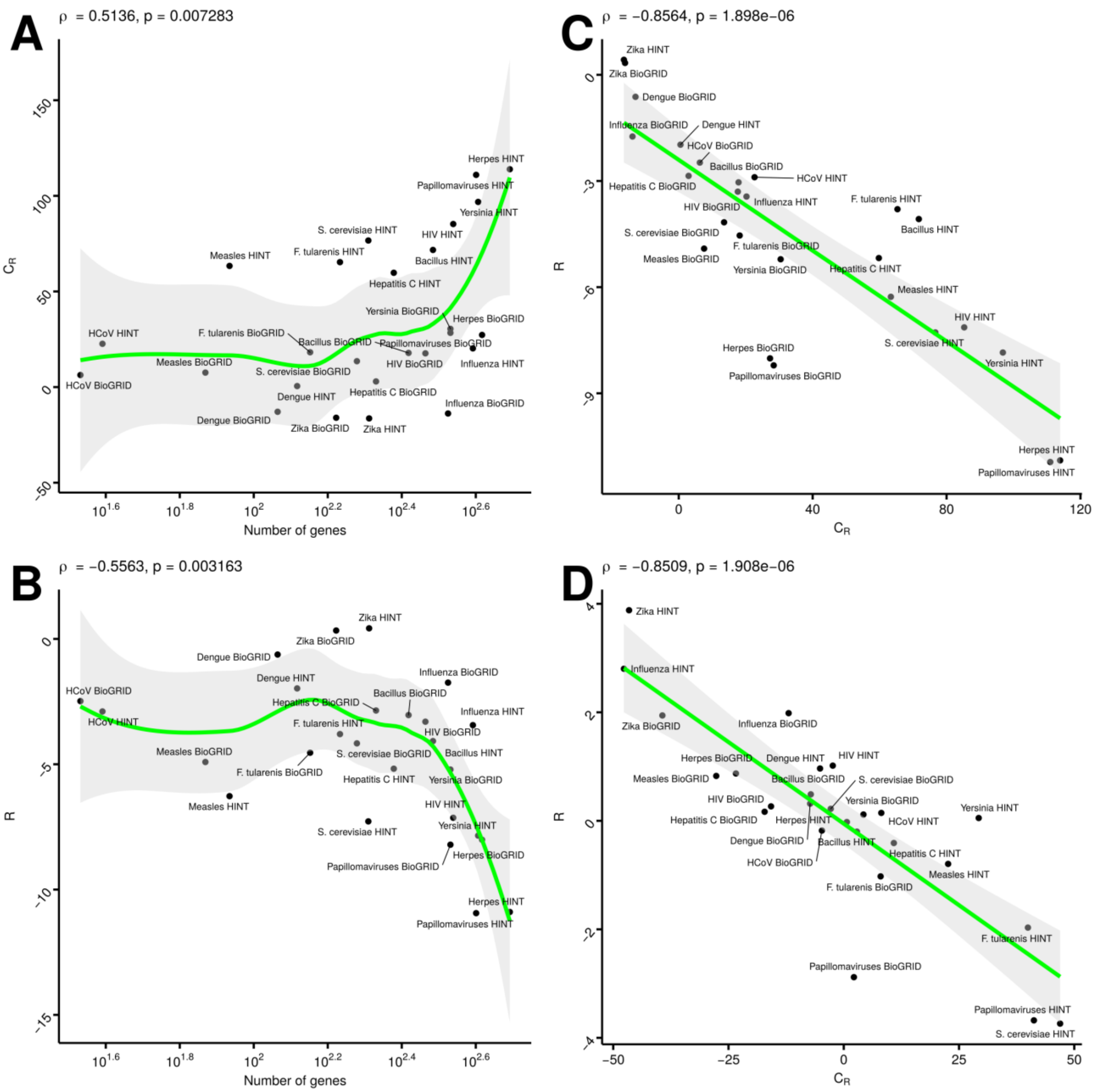

Supplementary Figure S9: Redundancy and robustness versus number of pathogen-related genes in the human multiplex against *RanDP-noC$_D$* null model. A) C_R for pathogen-related genes (against *RanDP-noC$_D$* null model) versus number of pathogen genes. B) R for pathogen-related genes (against *RanDP-noC$_D$* null model) versus number of pathogen genes. C) R versus C_R for pathogen-related genes against *RanDP-noC$_D$* null model. D) R versus C_R for pathogen-related genes while controlling for number of genes. R and C_R are regressed using loess regression against number of genes and residuals are plotted. Pathogen names are annotated with the PPI database used for the analysis. For all the panels, Spearman rank correlation values, ρ, are also listed along with p-values (two-tailed z-test using Fisher's z-transformation) are also shown.

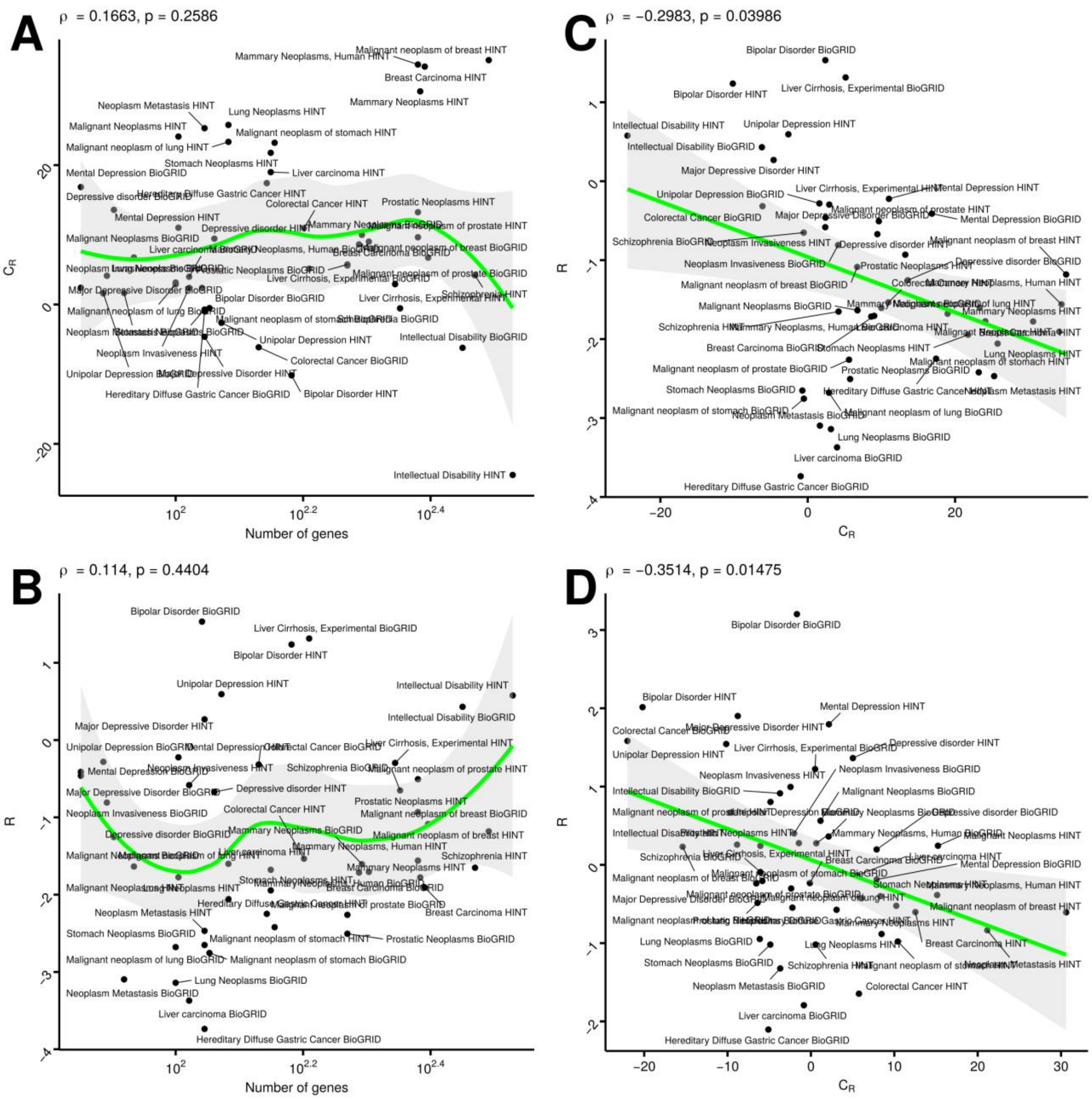

Supplementary Figure S10: Redundancy and robustness versus number of disease-related genes in the human multiplex against *RanDP* null model. A) C_R for disease-related genes (against *RanDP* null model) versus number of disease genes. B) R for disease-related genes (against *RanDP* null model) versus number of disease genes. C) R versus C_R for disease-related genes (against *RanDP* null model). D) R versus C_R for disease-related genes while controlling for number of genes. R and C_R are regressed using loess regression against number of genes and residuals are plotted. Disease names are annotated with the PPI database used for the analysis. For all the panels, Spearman rank correlation values, ρ, are also listed along with p-values (two-tailed z-test using Fisher's z-transformation) are also shown.

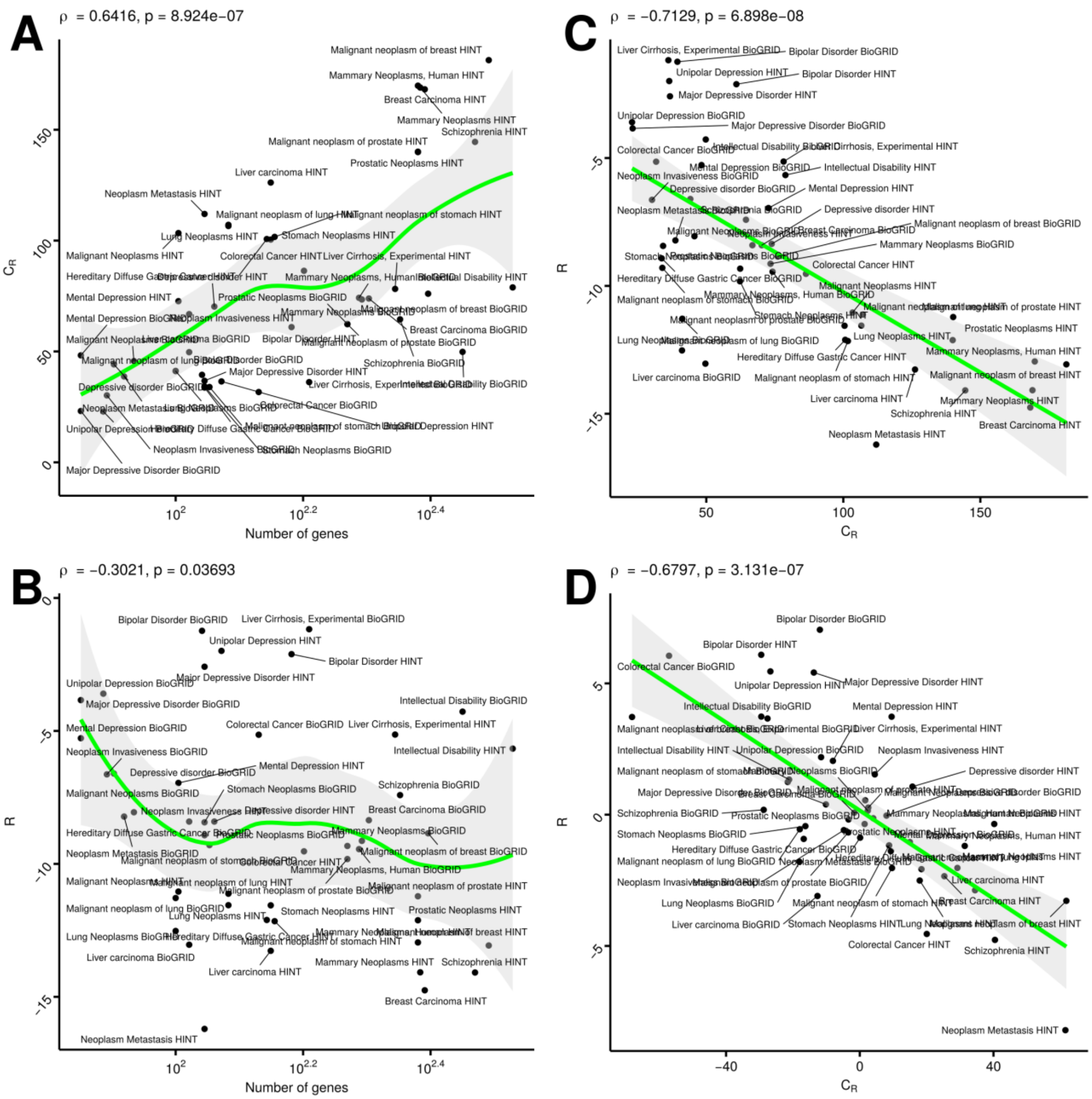

Supplementary Figure S11: Redundancy and robustness versus number of disease-related genes in the human multiplex against *RanDP-RZ* null model. A) C_R for disease-related genes (against *RanDP-RZ* null model) versus number of disease genes. B) R for disease-related genes (against *RanDP-RZ* null model) versus number of disease genes. C) R versus C_R for disease-related genes (against *RanDP-RZ* null model). D) R versus C_R for disease-related genes while controlling for number of genes. R and C_R are regressed using loess regressions against number of genes and residuals are plotted. Disease names are annotated with the PPI database used for the analysis. For all the panels, Spearman rank correlation values, ρ, are also listed along with p-values (two-tailed z-test using Fisher's z-transformation) are also shown.

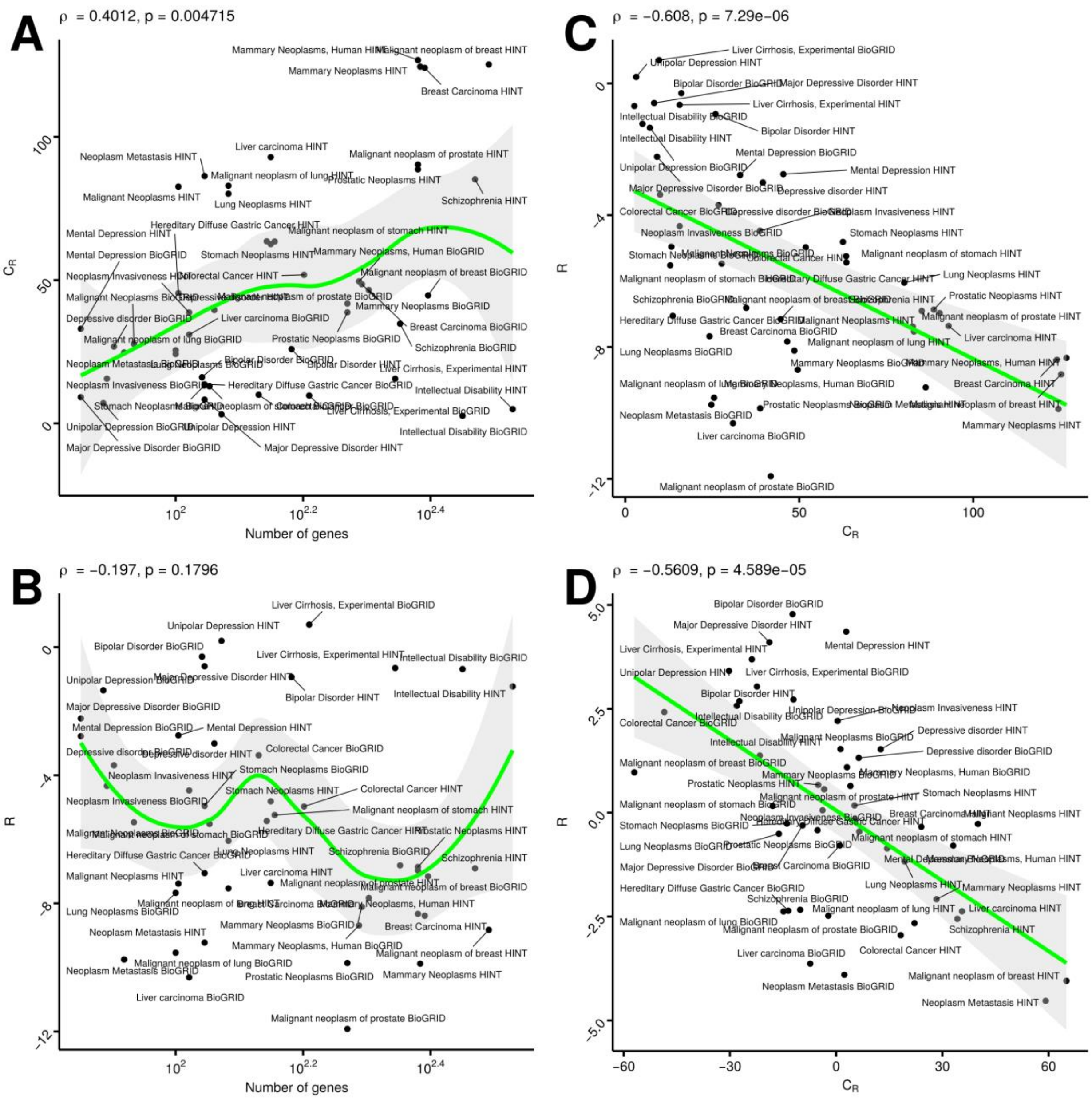

Supplementary Figure S12: Redundancy and robustness versus number of disease-related genes in the human multiplex against *RanDP-noC$_D$* null model. A) C_R for disease-related genes (against *RanDP-noC$_D$* null model) versus number of disease genes. B) R for disease-related genes (against *RanDP-noC$_D$* null model) versus number of disease genes. C) R versus C_R for disease-related genes (against *RanDP-noC$_D$* null model). D) R versus C_R for disease-related genes while controlling for number of genes. R and C_R are regressed using loess regression against number of genes and residuals are plotted. Disease names are annotated with the PPI database used for the analysis. For all the panels, Spearman rank correlation values, ρ, are also listed along with p-values (two-tailed z-test using Fisher's z-transformation) are also shown.

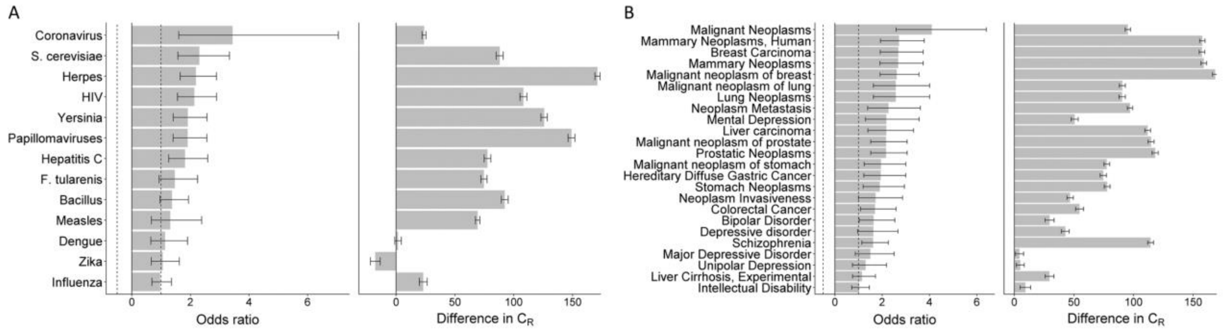

Supplementary Figure S13: Disease- and pathogen-related genes are enriched in the human multiplex. A) (Left) Odds ratio between pathogen and non-pathogen genes for different pathogens. (Right) difference in redundancy coupling (C_R) between pathogen and non-pathogen genes. B) (Left) Odds ratio between disease and non- disease genes for different diseases. (Right) difference in redundancy coupling (C_R) between disease and non- disease genes. In all the panels, error bars show 95% confidence intervals.

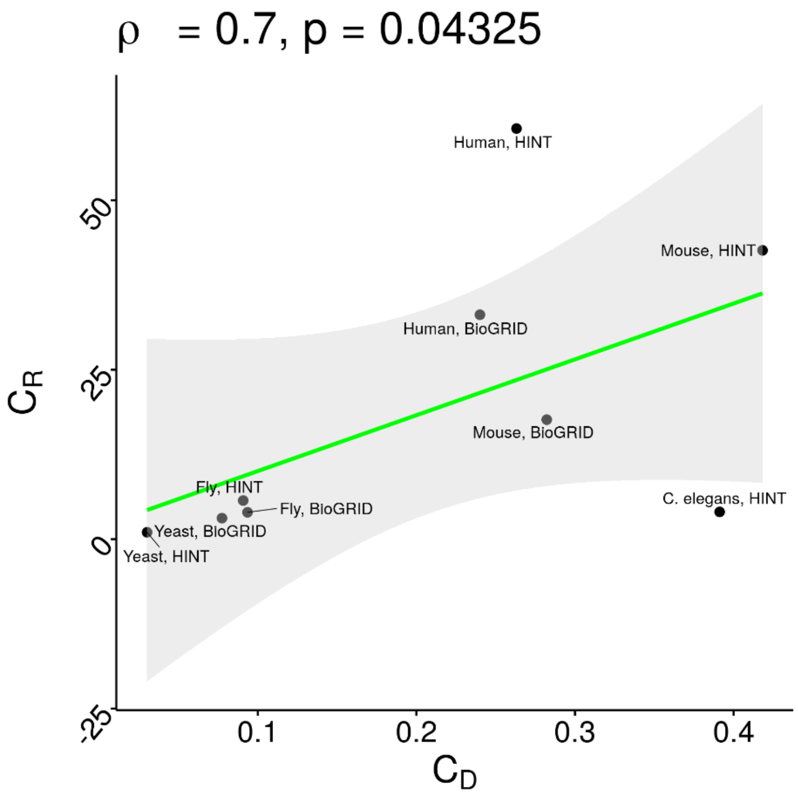

Supplementary Figure S14: Relationship between degree-degree and redundancy couplings across species. Scatter plot between redundancy coupling (C_R) and degree-degree coupling (C_D). C_R and C_D are correlated with a Pearson correlation of 0.7 (p = 0.043). P-values are calculated with a two-tailed z-test using Fisher's z-transformation.

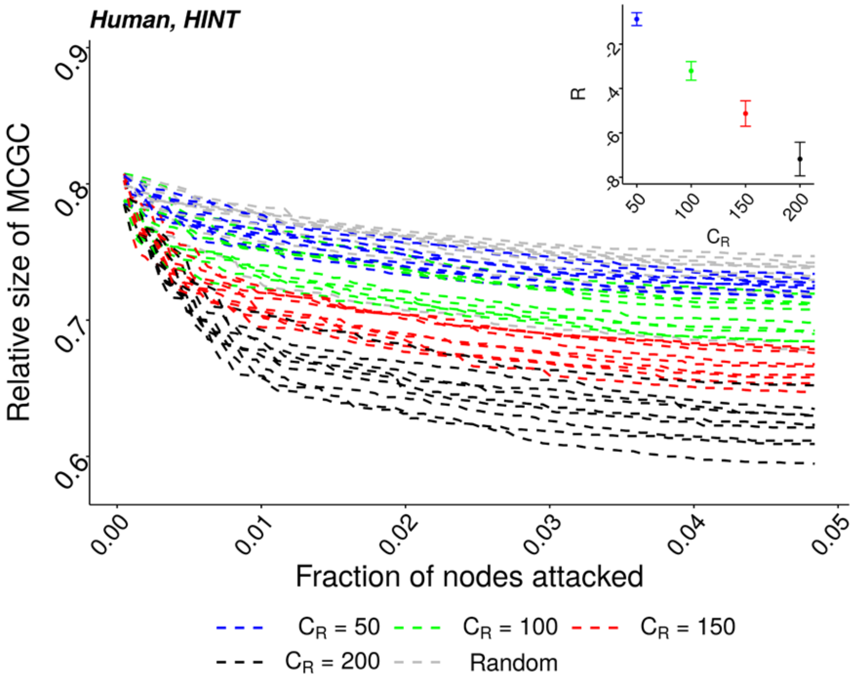

Supplementary Figure S15: Simulations showing dependence of robustness on redundancy coupling. We sample 100 gene-protein pairs from the human multiplex. We performed the sampling experiment multiple times, each time generating a set with a different value of redundancy coupling (C_R). 'Random' refers to a set of randomly sampled gene-protein pairs. For each value of C_R, sampling was repeated 100 times. Partial attack curves for gene-protein pairs sampled from the human multiplex with different values of C_R are shown. Increasing C_R reduces area under the attack curve and also reduces robustness (inset). Error bars show 95% confidence intervals. HINT PPI network was used for the simulations.

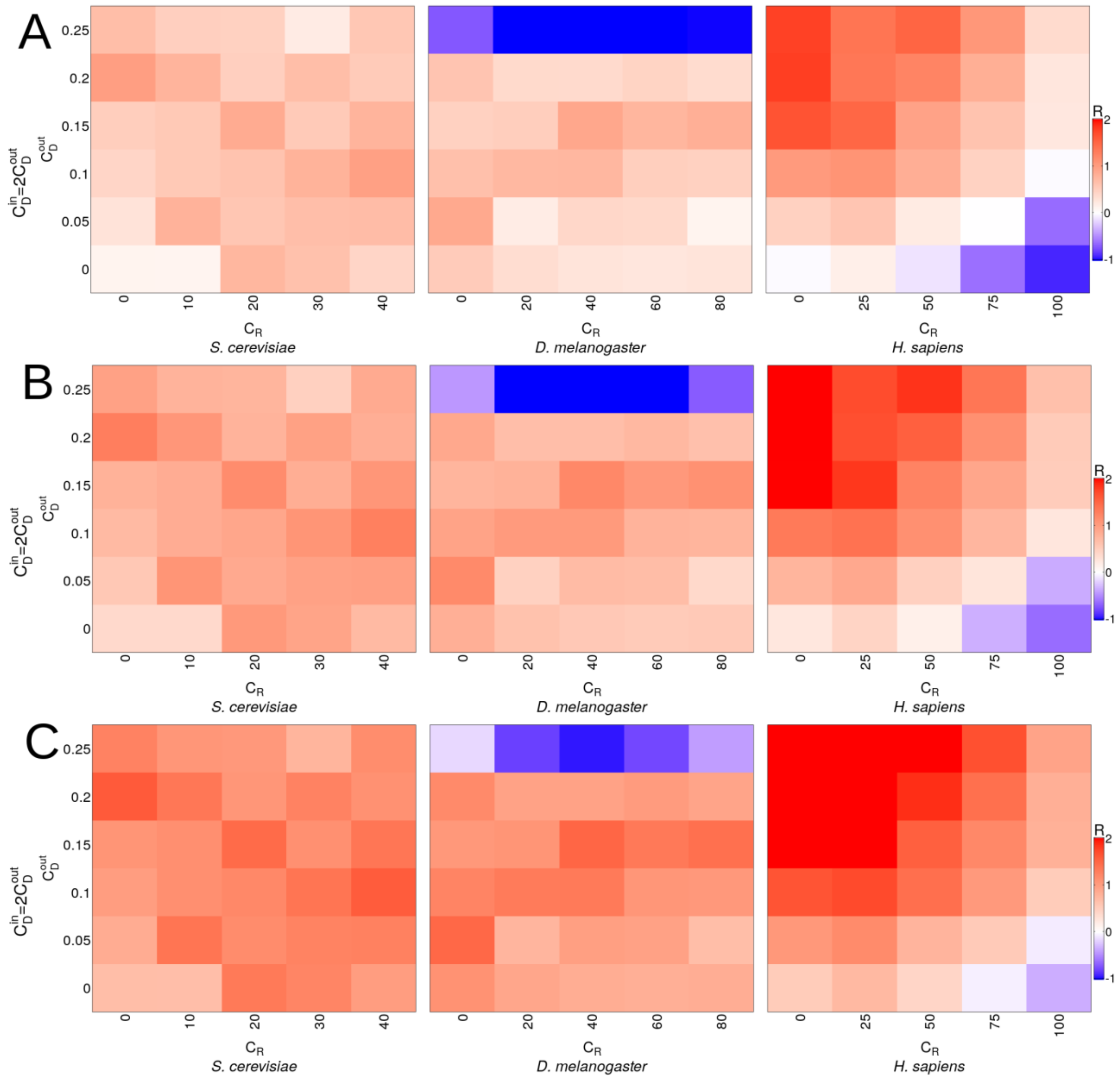

Supplementary Figure S16: Impact of size of multiplex on robustness. We compared robustness between two subsets of gene-protein pairs, with different sizes, extracted from the same species multiplex over a grid of values for C_D and C_R. For each pair of C_D and C_R values over the grid, we sample subsets of two different sizes for each species such that C_D and C_R values are the same for the two sampled subsets. Each cell in the heatmap shows relative robustness as the cohen's d difference between the RobustAreas of the larger and smaller subsets with the same C_D and C_R values. The sizes of the two subsets for each species are: *S. cerevisiae*-1000 and 1250, *D. melanogaster*-2000 and 2500, *H. sapiens*-500 and 750. For each subset, sampling was performed 100 times. A), B) and C) represent the mean, lower limit for the 95% CI and upper limit for the 95% CI for relative robustness between the two subsets. All panels use the BioGRID PPI.

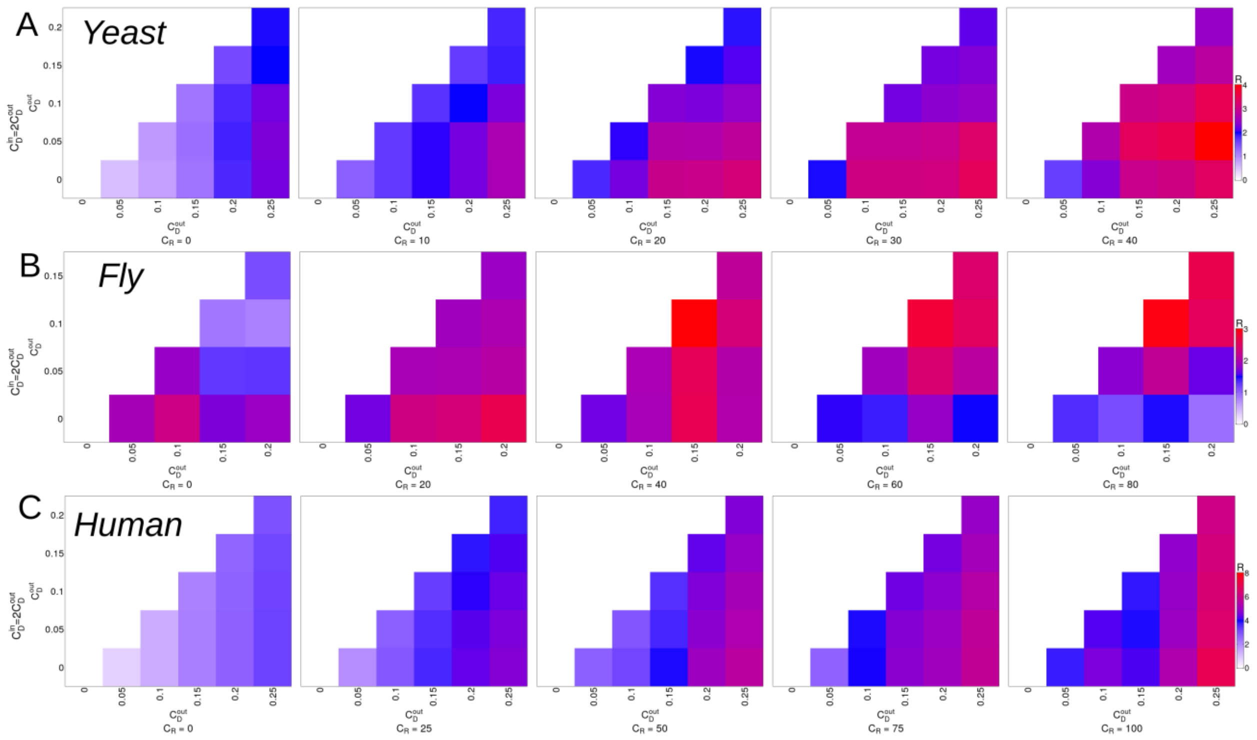

Supplementary Figure S17: Comparison between multiplexes of different sizes can reveal the impact of degree-degree coupling on robustness. We compared robustness between two subsets of gene-protein pairs, with different sizes, extracted from the same species multiplex over a grid of values for C_D for the two subsets. Values on the y-axis are the C_D values for the smaller subset against which we compare robustness of the larger subset. C_D values for the larger subset are shown on the x-axis. For each pair of C_D values over the grid, the smaller sized subset has C_D value shown on the y-axis, while the larger subset has C_D value shown on the x-axis. Each heatmap corresponds to one value of C_R, which is same for both the subsets. Each cell in the heatmap shows relative robustness as the cohen's d difference between the RobustAreas of the larger and smaller subsets. The sizes of the two subsets for each species are: *S. cerevisiae*-1000 and 1250, *D. melanogaster*-2000 and 2500, *H. sapiens*-500 and 750. For each subset, sampling was performed 100 times. Moving along the x-axis while keeping the y-axis value fixed, we see that robustness (R) increases with increasing C_D of the larger sized multiplex. For each row in a heatmap, R is computed by comparing the larger subset against the smaller subset. All panels use the BioGRID PPI. A), B) and C) show heatmaps for *S. cerevisiae*, *D. melanogaster* and *H. sapiens* respectively. Only the lower triangular part of the matrix is shown.

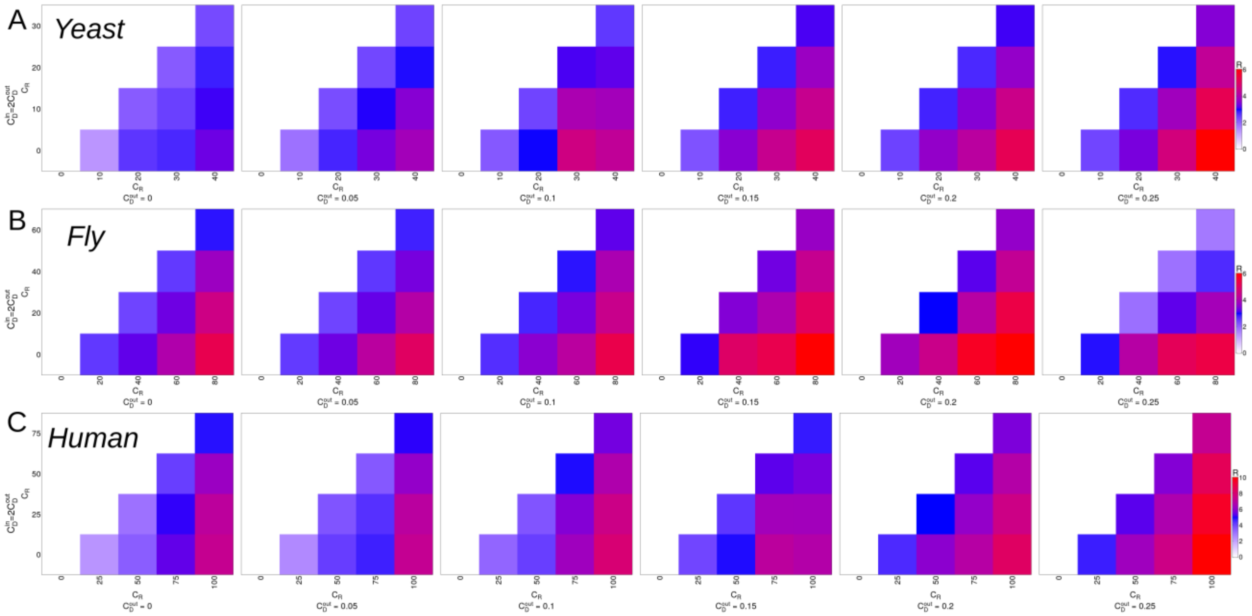

Supplementary Figure S18: Comparison between multiplexes of different sizes can reveal the impact of redundancy coupling on robustness. We compared robustness between two subsets of gene-protein pairs, with different sizes, extracted from the same species multiplex over a grid of values for C_R for the two subsets. Values on the y-axis are the C_R values for the smaller subset against which we compare robustness of the larger subset. C_R values for the larger subset are shown on the x-axis. For each pair of C_R values over the grid, the smaller sized subset has C_R value shown on the y-axis, while the larger subset has C_R value shown on the x-axis. Each heatmap corresponds to one value of C_D, which is same for both the subsets. Each cell in the heatmap shows relative robustness as the cohen's d difference between the RobustAreas of the larger and smaller subsets. The sizes of the two subsets for each species are: *S. cerevisiae*-1000 and 1250, *D. melanogaster*-2000 and 2500, *H. sapiens*-500 and 750. For each subset, sampling was performed 100 times. Moving along the x-axis while keeping the y-axis value fixed, we see that robustness (R) increases with increasing C_R of the larger sized multiplex. For each row in a heatmap, R is computed by comparing the larger subset against the smaller subset. All panels use the BioGRID PPI. A), B) and C) show heatmaps for *S. cerevisiae*, *D. melanogaster* and *H. sapiens* respectively. Only the lower triangular part of the matrix is shown.

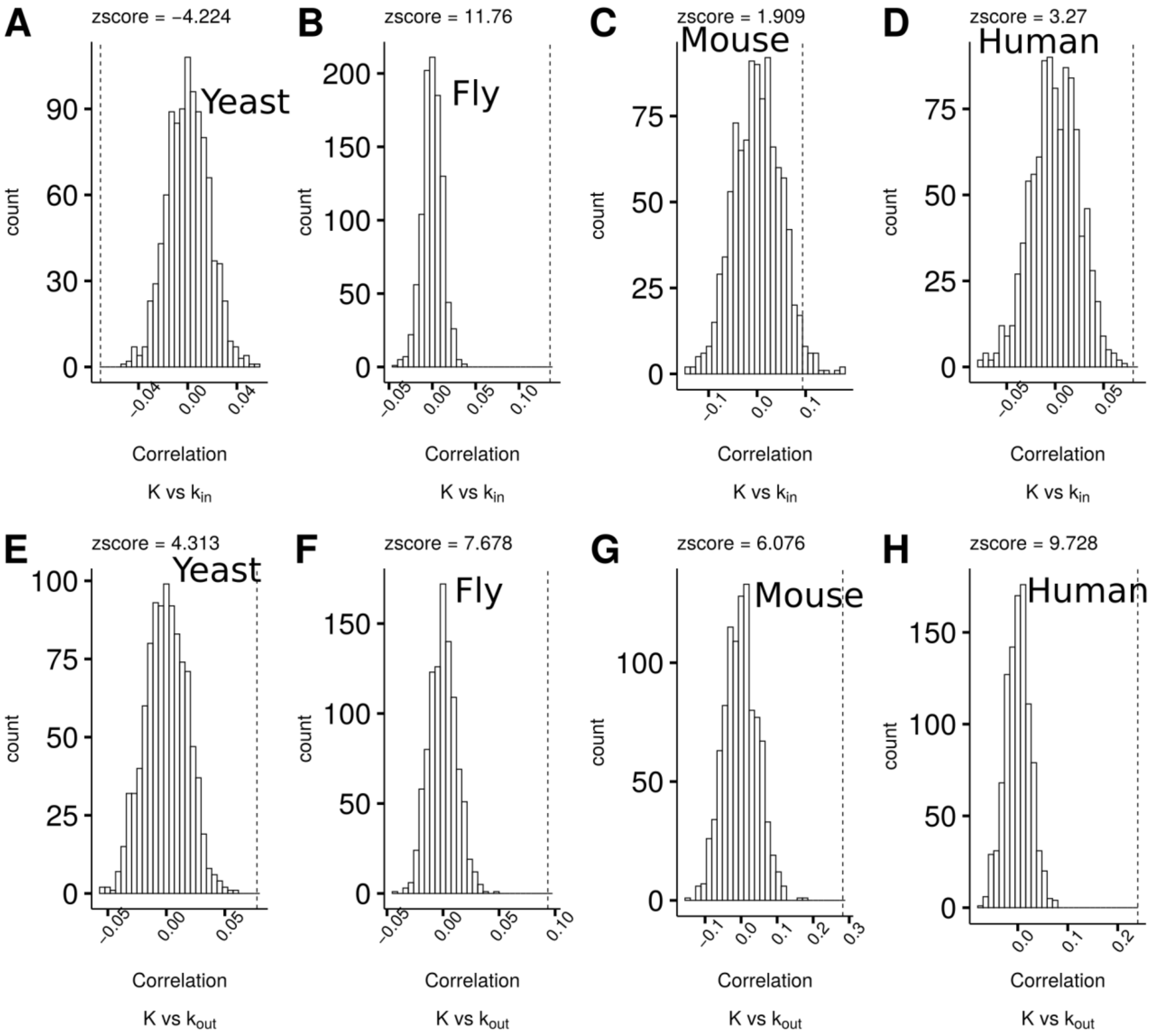

Supplementary Figure S19: Comparing species degree-degree coupling against randomly shuffled multiplexes for BioGRID PPI. We compared species degree-degree coupling (dashed line) against the distribution (histogram) of degree-degree coupling for randomly shuffled multiplexes (PPI fixed and gene labels randomly shuffled in TRN). Z-score values show that species degree-degree couplings are statistically significant.

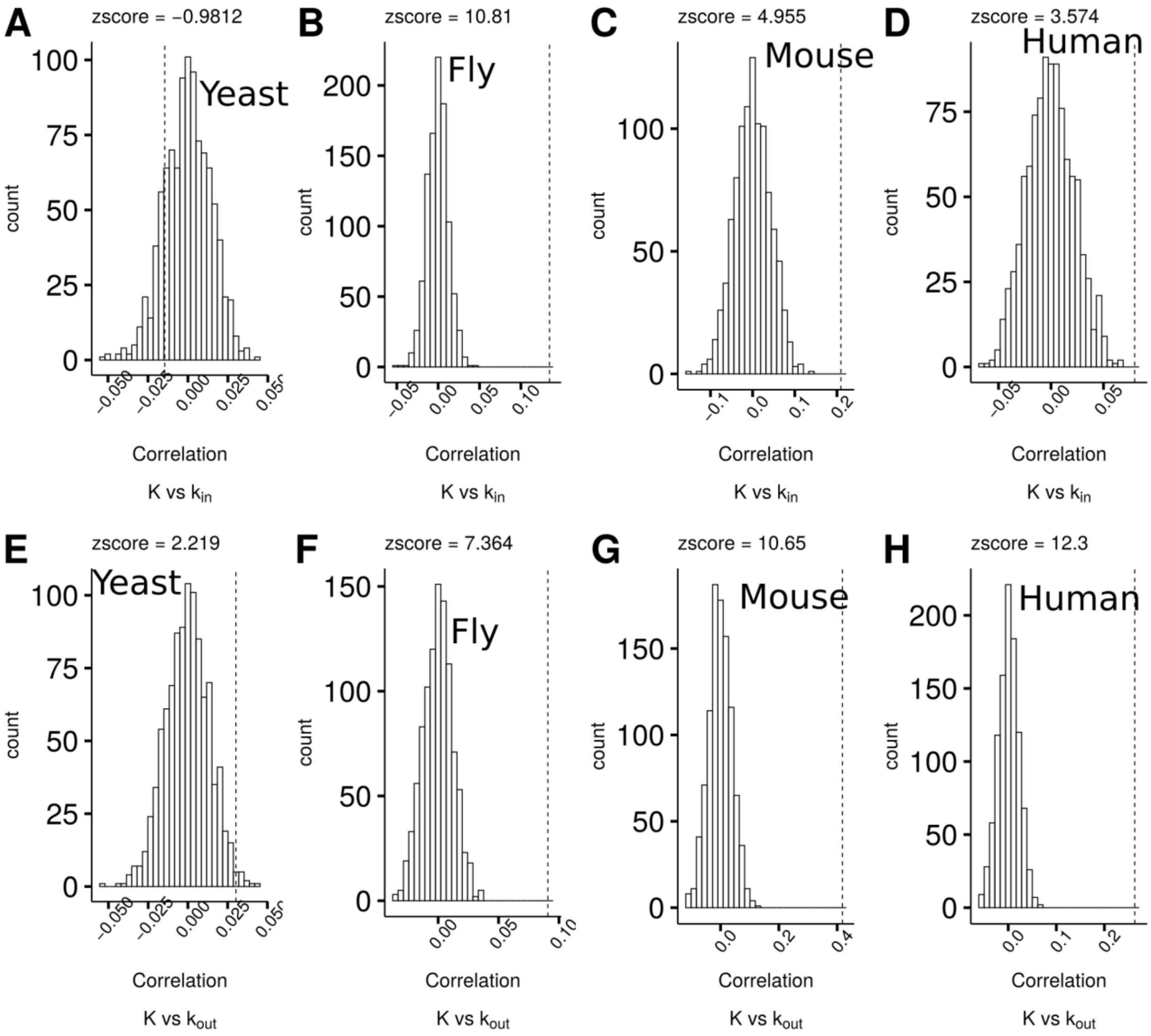

Supplementary Figure S20: Comparing species degree-degree coupling against randomly shuffled multiplexes for HINT PPI. We compared species degree-degree coupling (dashed line) against the distribution (histogram) of degree-degree coupling for randomly shuffled multiplexes (PPI fixed and gene labels randomly shuffled in TRN). Z-score values show that species degree-degree couplings are statistically significant.

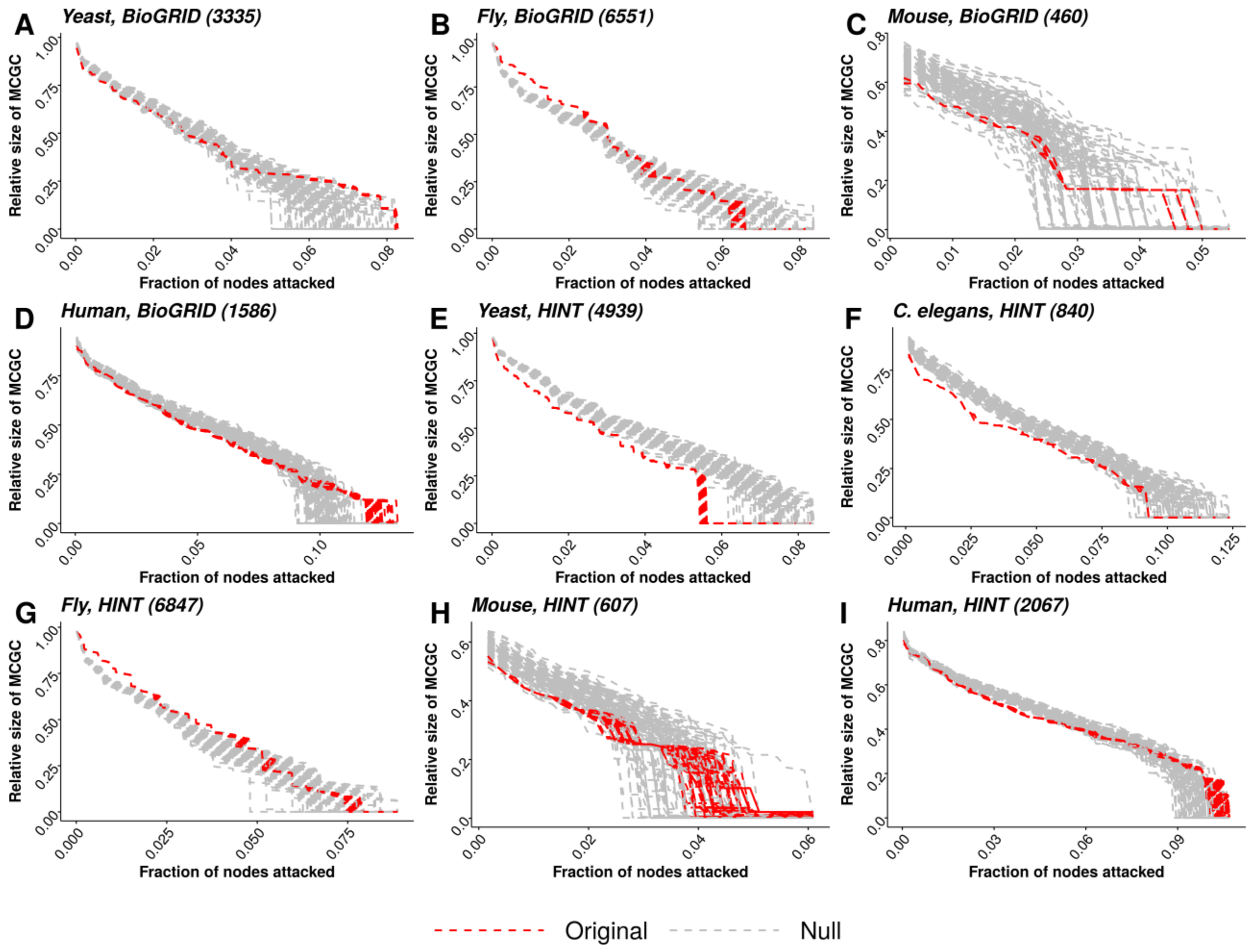

Supplementary Figure S21: Multiplex attack curves for different species against the *Multiplex-Configuration* null model. Relative size of Mutually Connected Giant Component (MCGC) is plotted as a function of the fraction of gene-protein pairs attacked and removed from the multiplex (Main Text, Methods). Attack curves are shown for five species using PPI networks from either BioGRID or HINT databases (Main Text, Methods); database for a given panel are annotated next to the species name. Along with the attack curves for the species (red), attack curves for the *Multiplex-Configuration* null model are also shown (gray). Under this null model, gene and protein degrees in TRN and PPI are kept fixed, while edges are randomly shuffled. Further, the one-to-one correspondence between TRN genes and PPI proteins is kept fixed.

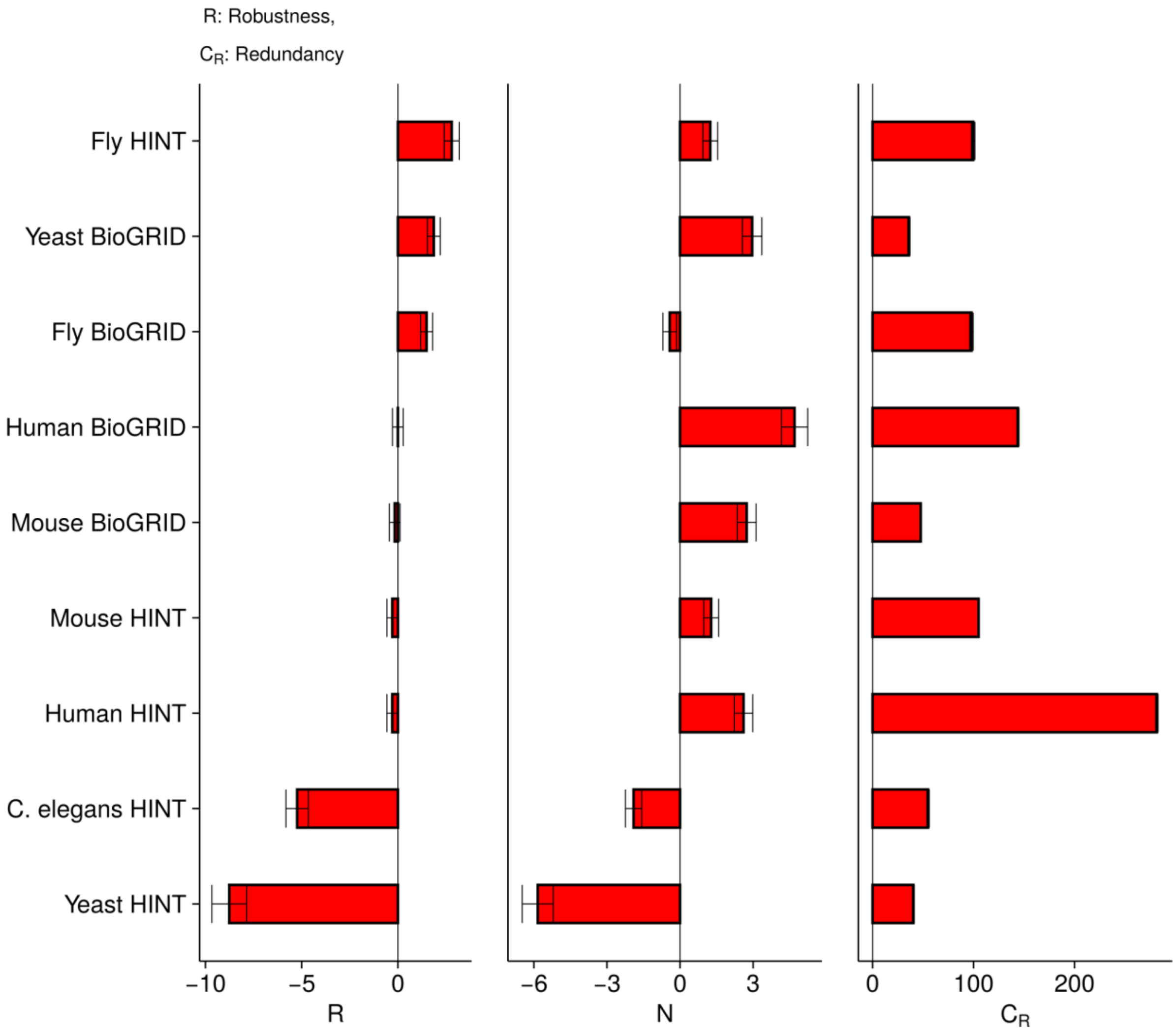

Supplementary Figure S22: Degree-degree and redundancy coupling for species against the Multiplex-Configuration null model. (Left) Robustness (R) to targeted attack against the configuration null model. (Middle) Size robustness (rather than RobustArea, RobustSize is used for robustness. RobustSize is the number of gene-protein pairs that need to be attacked to break the multiplex) to targeted attack against the configuration null model. (Right) Degree-degree coupling against the configuration null model. Error bars show 95% CI.

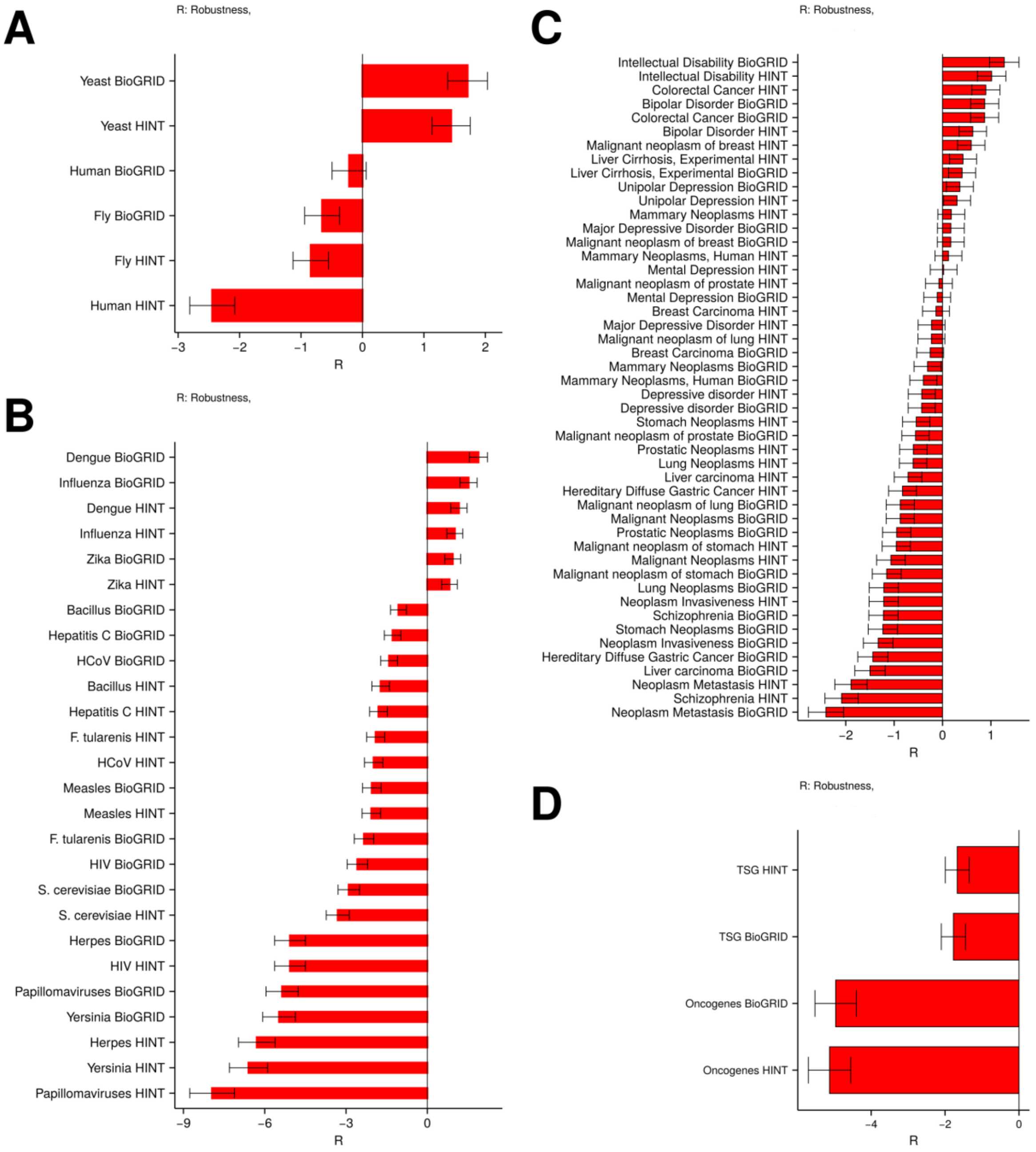

Supplementary Figure S23: Functionally important genes and proteins are topologically important in TRN against RanDP null model. A) Fly and human essential genes and proteins have lower robustness (R) to targeted attack in TRN against *RanDP* null model. However, yeast essential genes and proteins are more robust. Panels B-D show results for human multiplex. B) Pathogen-related genes and proteins have lower R to targeted attack in TRN for most pathogens against *RanDP* null model. C) Disease-related genes and proteins have lower R to targeted attack for most diseases against *RanDP* null model. D) Oncogenes (tumor suppressor genes (TSGs)) and proteins have lower R to targeted attack against *RanDP* null model. In all the panels, database used for PPI networks is annotated on the y-axis. In all the panels, relative robustness (R) is measured against *RanDP* null model. Error bars show 95% CIs.

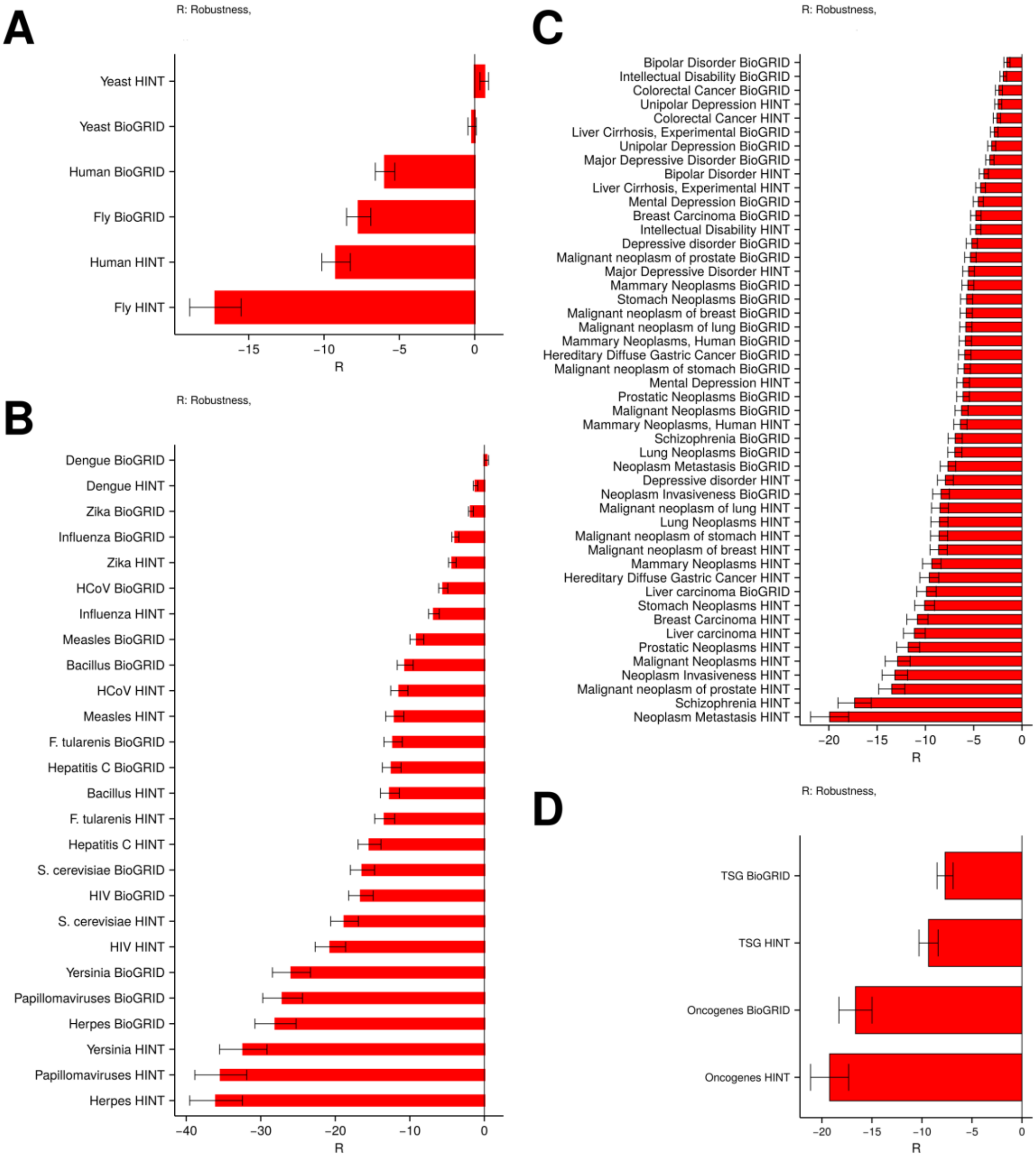

Supplementary Figure S24: Functionally important genes and proteins are topologically important in TRN against RanDP-RZ null model. A) Fly and human essential genes and proteins have lower robustness (R) to targeted attack in TRN against *RanDP-RZ* null model. However, yeast essential genes and proteins are indistinguishable from the null model in robustness. Panels B-D show results for human multiplex. B) Pathogen-related genes and proteins have lower R to targeted attack in TRN across pathogens against *RanDP-RZ* null model. C) Disease-related genes and proteins have lower R to targeted attack across diseases against *RanDP-RZ* null model. D) Oncogenes (tumor suppressor genes (TSGs)) and proteins have lower R to targeted attack against *RanDP-RZ* null model. In all the panels, database used for PPI networks is annotated on the y-axis. In all the panels, relative robustness (R) is measured against *RanDP-RZ* null model. Error bars show 95% CIs.

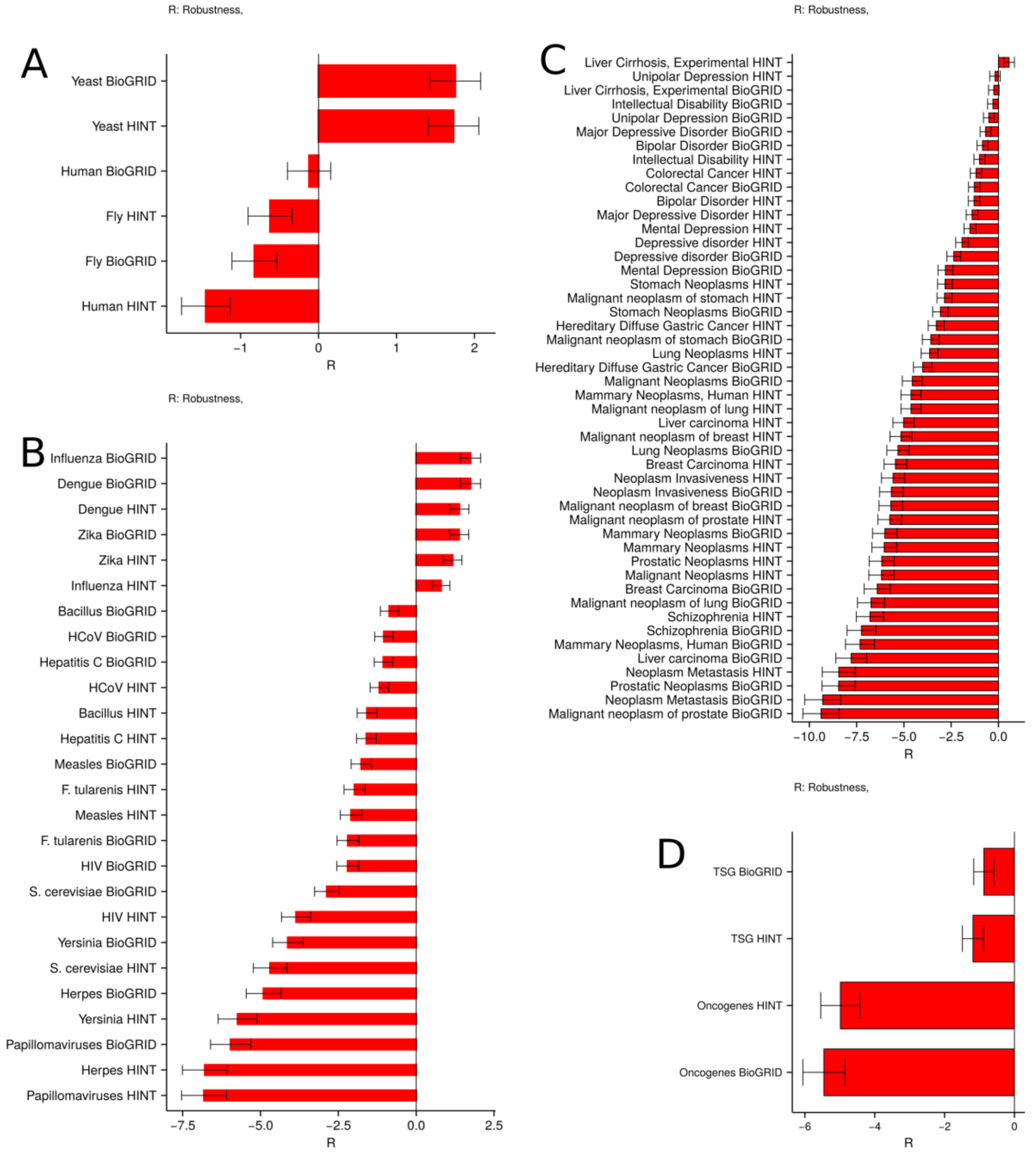

Supplementary Figure S25: Functionally important genes and proteins are topologically important in TRN against RanDP-noCD null model. A) Fly and human essential genes and proteins have lower robustness (R) to targeted attack in TRN against *RanDP-noC$_D$* null model. However, yeast essential genes and proteins are more robust. Panels B-D show results for human multiplex. B) Pathogen-related genes and proteins have lower R to targeted attack in TRN for most pathogens against *RanDP-noC$_D$* null model. C) Disease-related genes and proteins have lower R to targeted attack across diseases against *RanDP-noC$_D$* null model. D) Oncogenes (tumor suppressor genes (TSGs)) and proteins have lower R to targeted attack against *RanDP-noC$_D$* null model. In all the panels, database used for PPI networks is annotated on the y-axis. In all the panels, relative robustness (R) is measured against *RanDP-noC$_D$* null model. Error bars show 95% CIs.

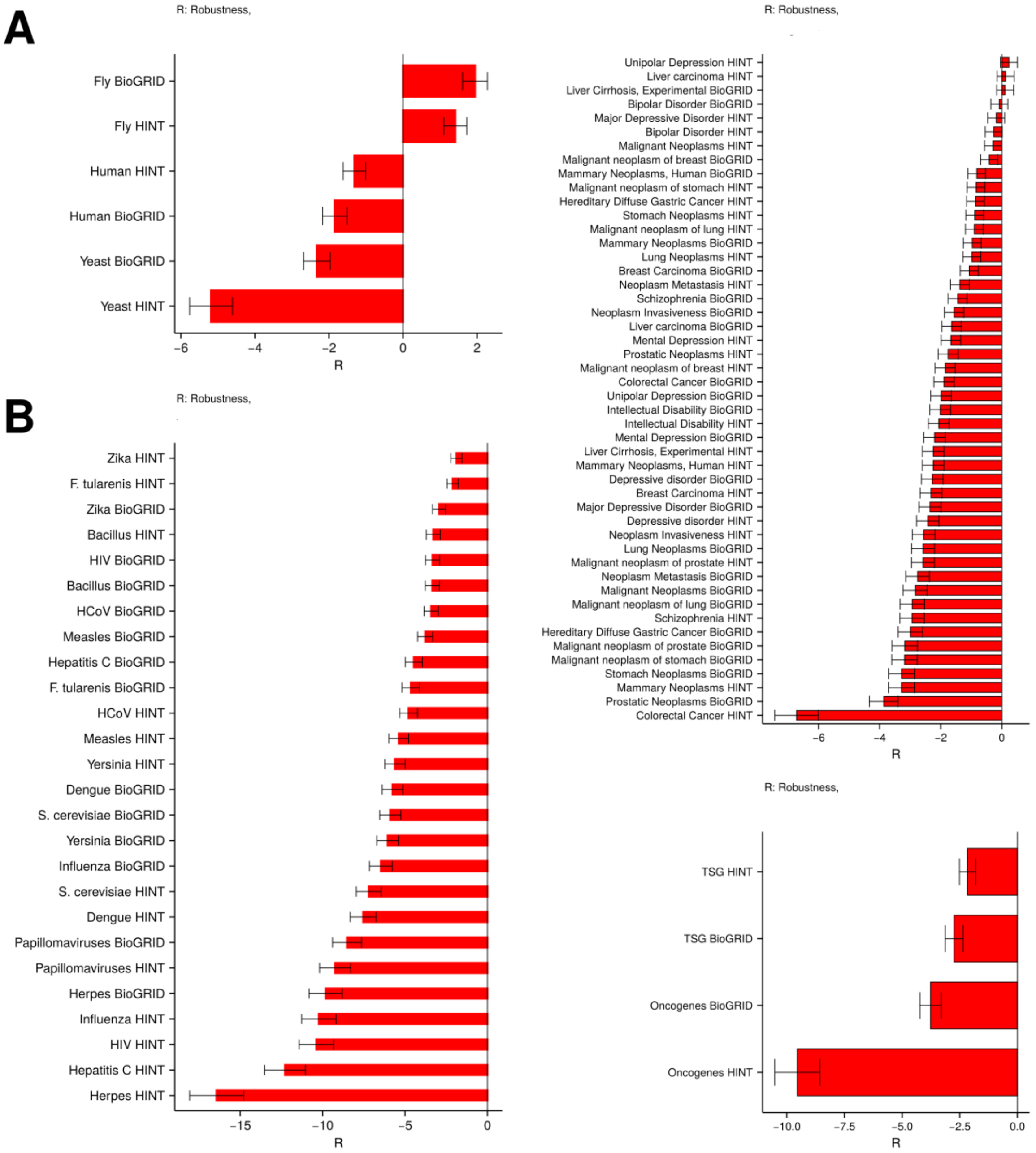

Supplementary Figure S26: Functionally important genes and proteins are topologically important in PPI against RanDP null model. A) Yeast and human essential genes and proteins have lower robustness (R) to targeted attack in PPI against *RanDP* null model. However, fly essential genes and proteins are more robust. Panels B-D show results for human multiplex. B) Pathogen-related genes and proteins have lower R to targeted attack in PPI across pathogens against *RanDP* null model. C) Disease-related genes and proteins have lower R to targeted attack for most diseases against *RanDP* null model. D) Oncogenes (tumor suppressor genes (TSGs)) and proteins have lower R to targeted attack against *RanDP* null model. In all the panels, database used for PPI networks is annotated on the y-axis. In all the panels, relative robustness (R) is measured against *RanDP* null model. Error bars show 95% CIs.

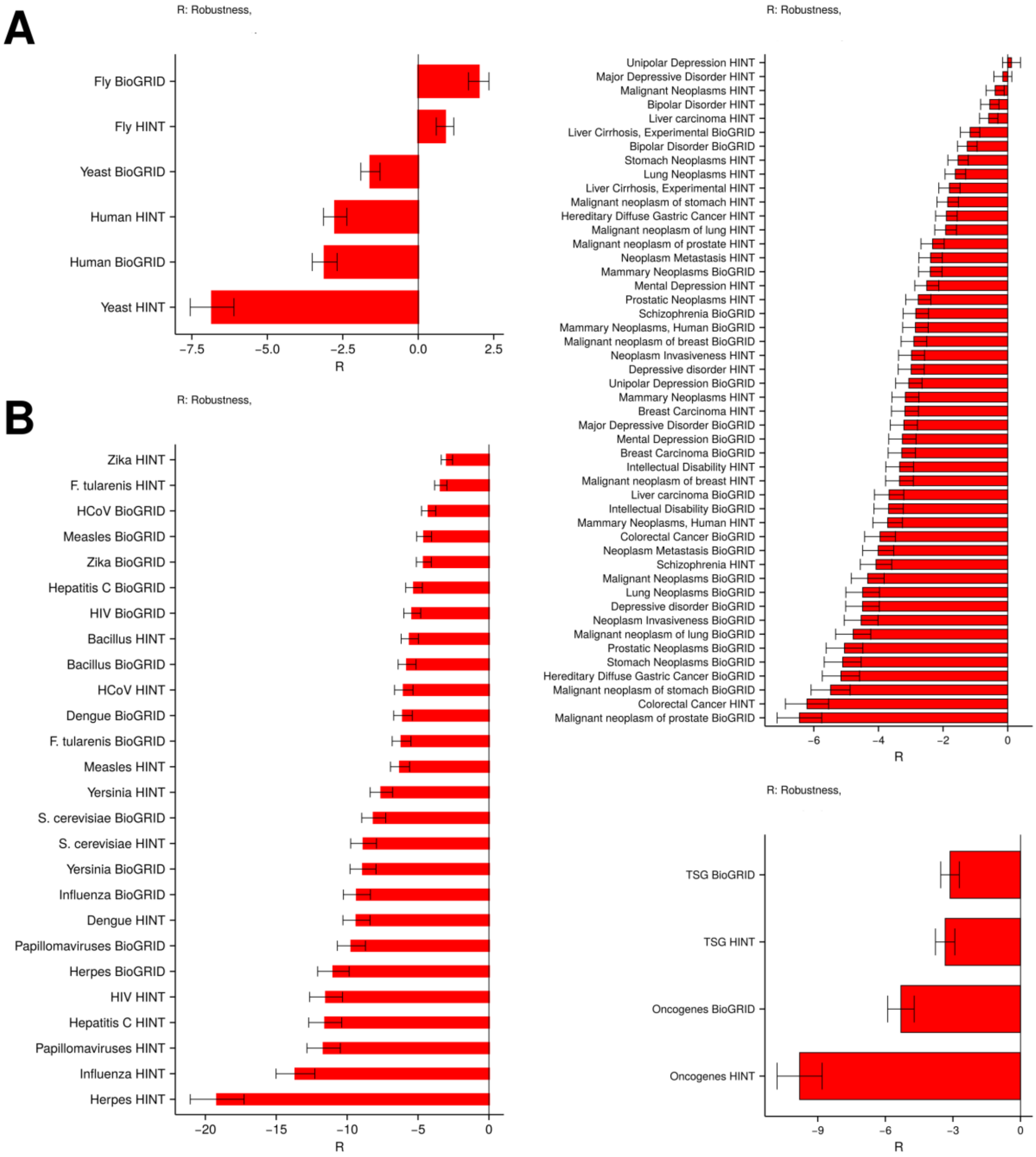

Supplementary Figure S27: Functionally important genes and proteins are topologically important in PPI against RanDP-RZ null model. A) Yeast and human essential genes and proteins have lower robustness (R) to targeted attack in PPI against *RanDP-RZ* null model. However, fly essential genes and proteins are more robust. Panels B-D show results for human multiplex. B) Pathogen-related genes and proteins have lower R to targeted attack in PPI across pathogens against *RanDP-RZ* null model. C) Disease-related genes and proteins have lower R to targeted attack for most diseases against *RanDP-RZ* null model. D) Oncogenes (tumor suppressor genes (TSGs)) and proteins have lower R to targeted attack against *RanDP-RZ* null model. In all the panels, database used for PPI networks is annotated on the y-axis. In all the panels, relative robustness (R) is measured against *RanDP-RZ* null model. Error bars show 95% CIs.

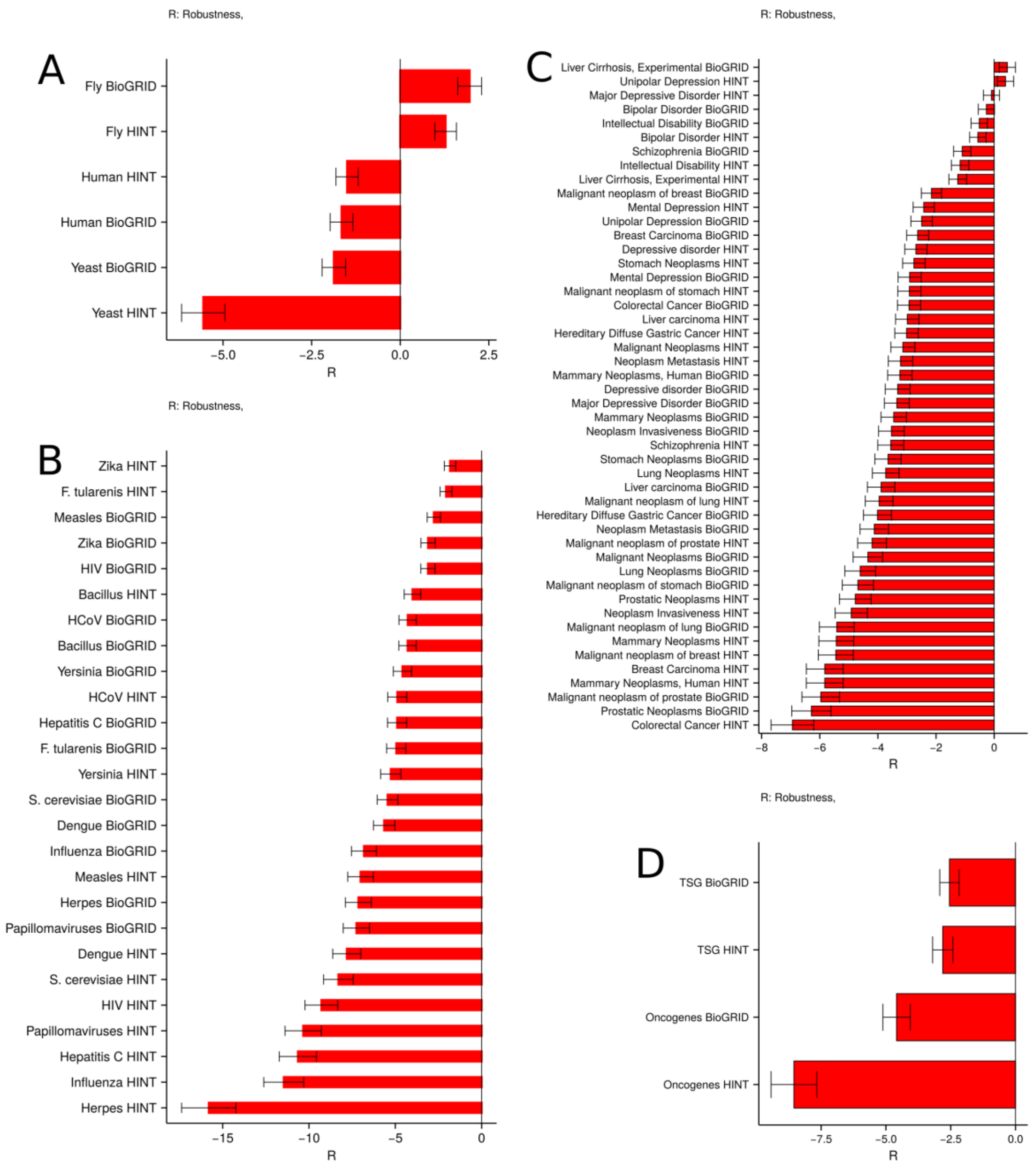

Supplementary Figure S28: Functionally important genes and proteins are topologically important in PPI against RanDP-noCD null model. A) Yeast and human essential genes and proteins have lower robustness (R) to targeted attack in PPI against *RanDP-noC*$_D$ null model. However, fly essential genes and proteins are more robust. Panels B-D show results for human multiplex. B) Pathogen-related genes and proteins have lower R to targeted attack in PPI across pathogens against *RanDP-noC*$_D$ null model. C) Disease-related genes and proteins have lower R to targeted attack for most diseases against *RanDP-noC*$_D$ null model. D) Oncogenes (tumor suppressor genes (TSGs)) and proteins have lower R to targeted attack against *RanDP-noC*$_D$ null model. In all the panels, database used for PPI networks is annotated on the y-axis. In all the panels, relative robustness (R) is measured against *RanDP-noC*$_D$ null model. Error bars show 95% CIs.

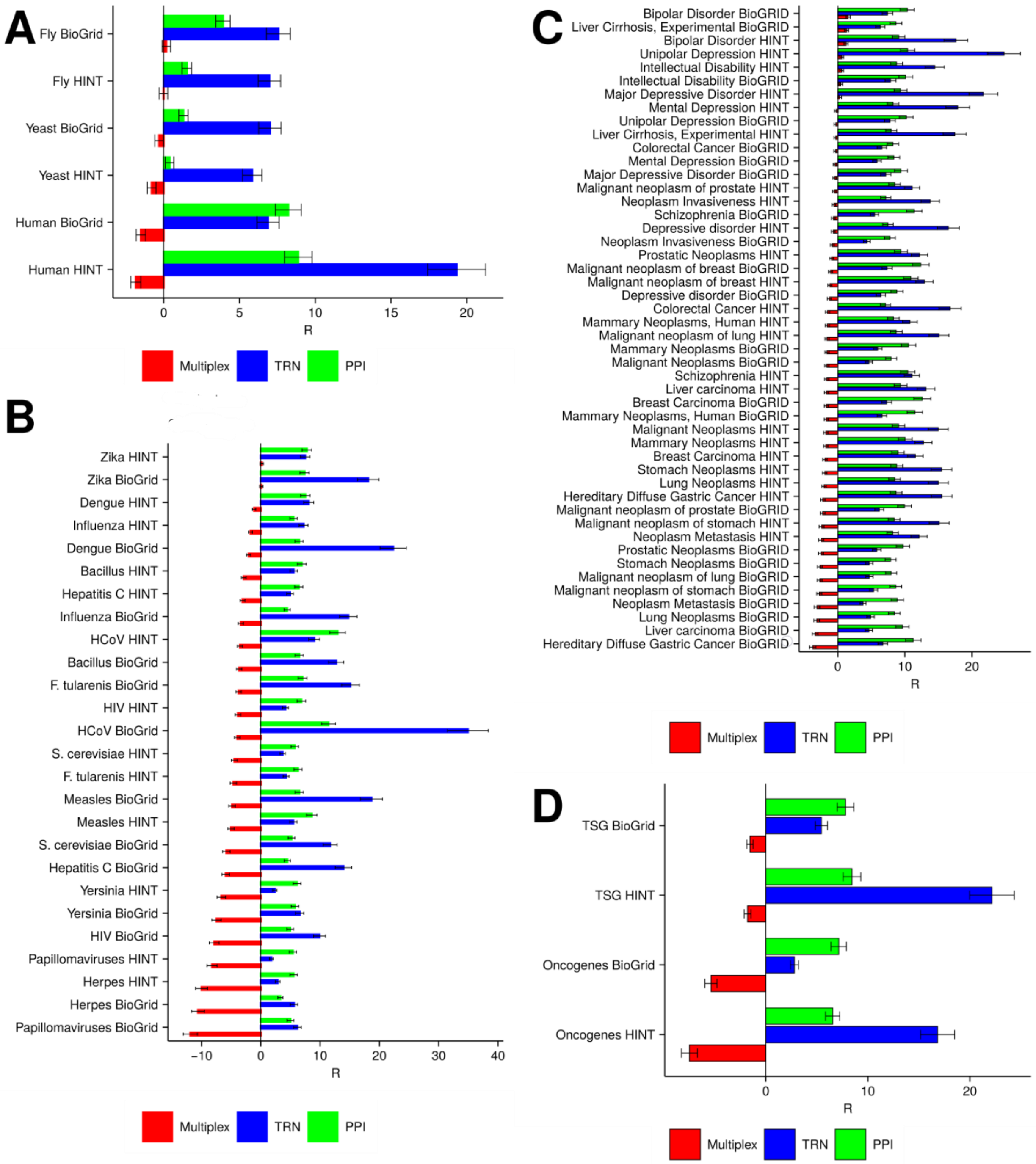

Supplementary Figure S29: Functionally important genes and proteins are topologically more vulnerable in species multiplex than TRN and PPI against *RanDP* null model. Across different functionally important genes and proteins--A) essential, B) pathogen-related, C) disease-related and D) oncogenes and TSGs—targeted attack on the multiplex is more vulnerable than independent targeted attack on either TRN or PPI. R is computed against attack on *RanDP* null multiplex. Panels B-D show results for the human multiplex. Error bars show 95% CI.

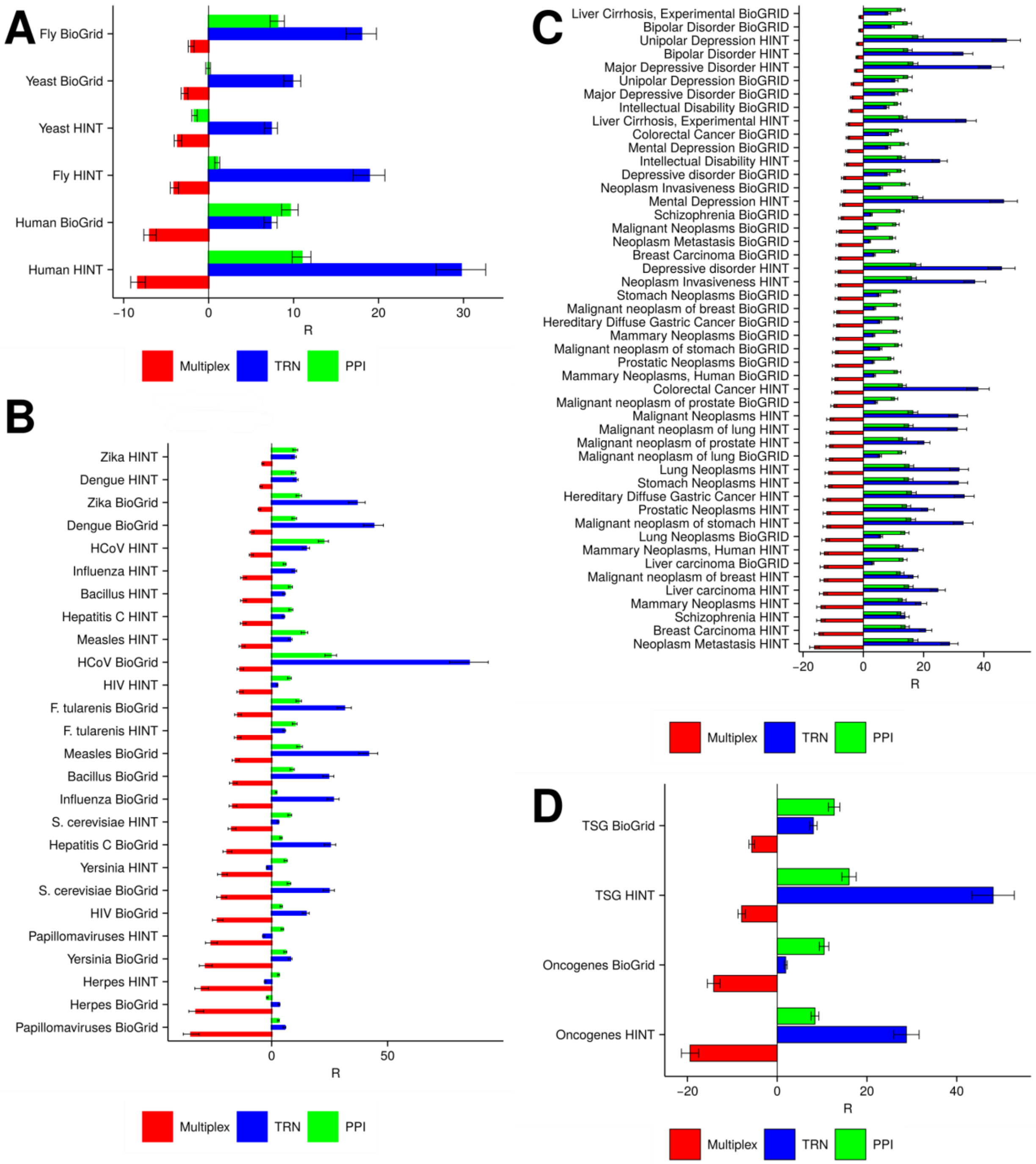

Supplementary Figure S30: Functionally important genes and proteins are topologically more vulnerable in species multiplex than TRN and PPI against *RanDP-RZ* null model. Across different functionally important genes and proteins--A) essential, B) pathogen-related, C) disease-related and D) oncogenes and TSGs—targeted attack on the multiplex is more vulnerable than independent targeted attack on either TRN or PPI. R is computed against attack on *RanDP-RZ* null multiplex. Panels B-D show results for the human multiplex. Error bars show 95% CI.

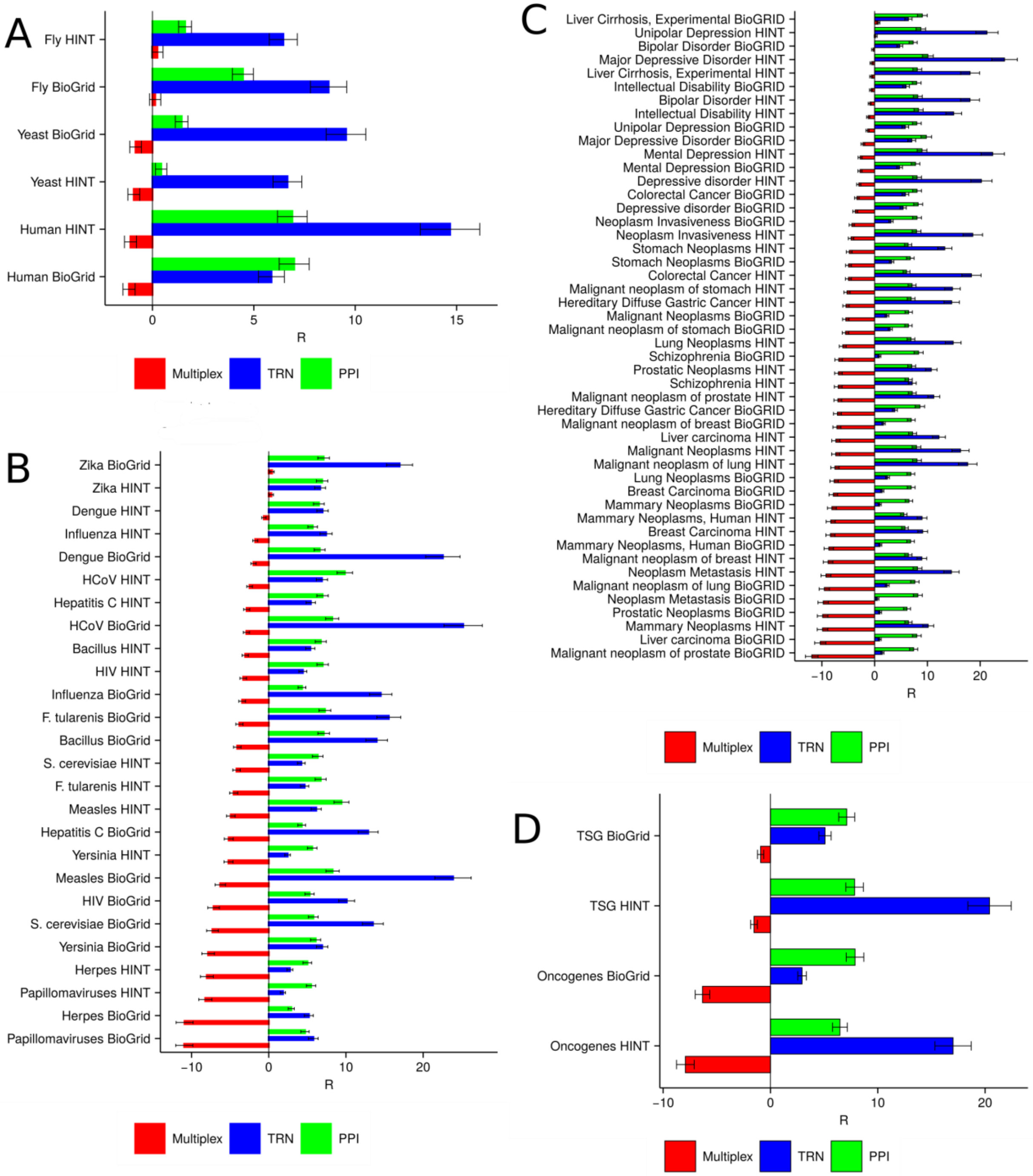

Supplementary Figure S31: Functionally important genes and proteins are topologically more vulnerable in species multiplex than TRN and PPI against *RanDP-noC*$_D$ null model. Across different functionally important genes and proteins--A) essential, B) pathogen-related, C) disease-related and D) oncogenes and TSGs—targeted attack on the multiplex is more vulnerable than independent targeted attack on either TRN or PPI. R is computed against attack on *RanDP-noC*$_D$ null multiplex. Panels B-D show results for the human multiplex. Error bars show 95% CI.

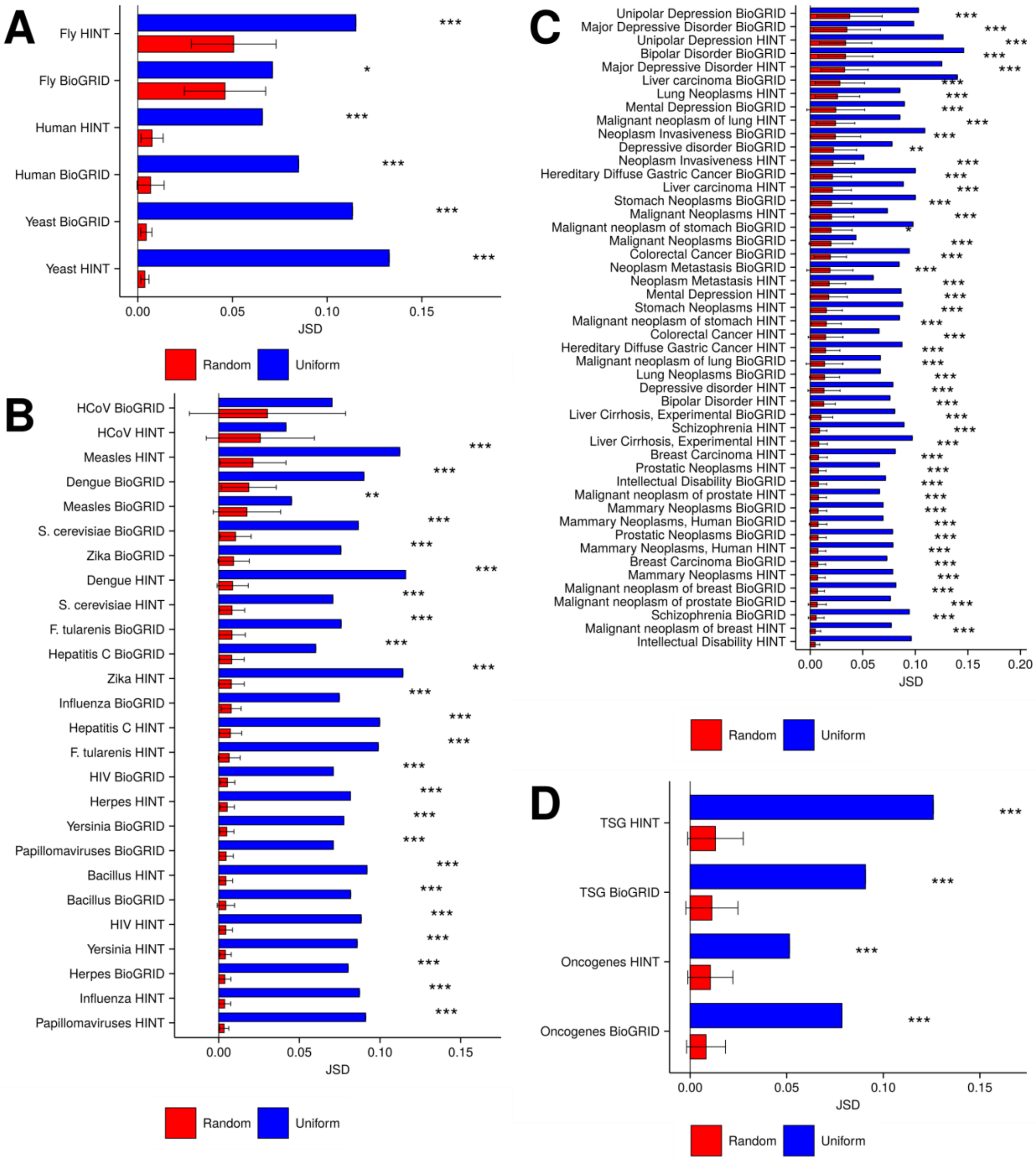

Supplementary Figure S32: Comparison between k_{in} distribution for functionally important genes and *RanDP* null model: Jensen Shannon Divergence (JSD) between k_{in} distributions for functionally important genes–A) essential, B) pathogen-related, C) disease-related and D) oncogenes and TSG–and *RanDP* null model sampled genes (red). As a control, we also show JSD between functionally important genes and uniform distribution (blue). JSD is computed by first binning k_{in} in the species multiplex such that each bin has at least 100 genes. Binned k_{in} values are used for JSD computation. Error bars show 95% CI. Statistical significance, for $\alpha = 0.05$, is marked by stars. Statistical significance is estimated using a two-tailed z-test without correction for multiple hypothesis testing.

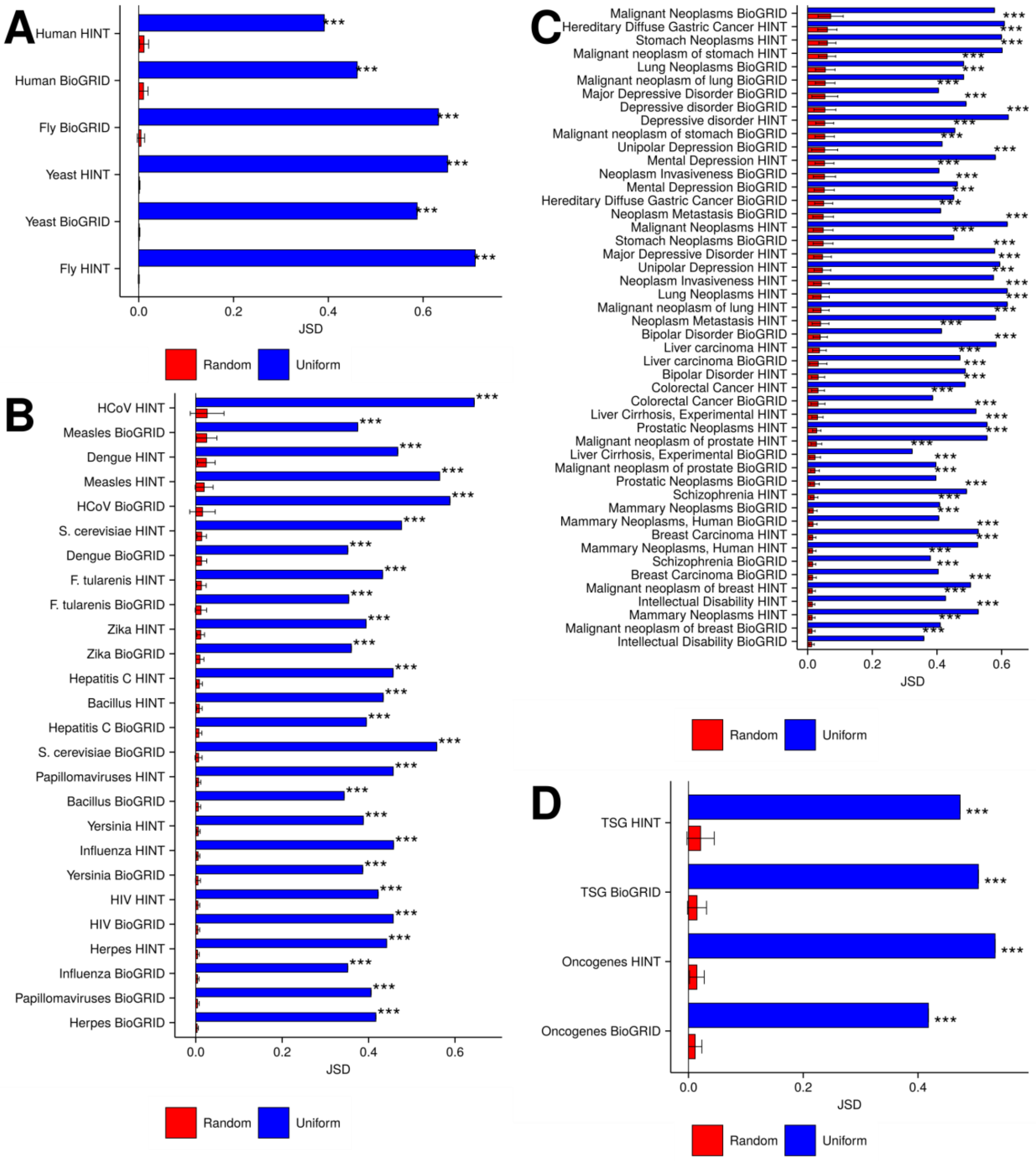

Supplementary Figure S33: Comparison between k_{out} distribution for functionally important genes and *RanDP* null model: Jensen Shannon Divergence (JSD) between k_{out} distributions for functionally important genes–A) essential, B) pathogen-related, C) disease-related and D) oncogenes and TSG–and *RanDP* null model sampled genes (red). As a control, we also show JSD between functionally important genes and uniform distribution (blue). JSD is computed by first binning k_{out} in the species multiplex such that each bin has at least 30 genes. Binned k_{out} values are used for JSD computation. Error bars show 95% CI. Statistical significance, for $\alpha = 0.05$, is marked by stars. Statistical significance is estimated using a two-tailed z-test without correction for multiple hypothesis testing.

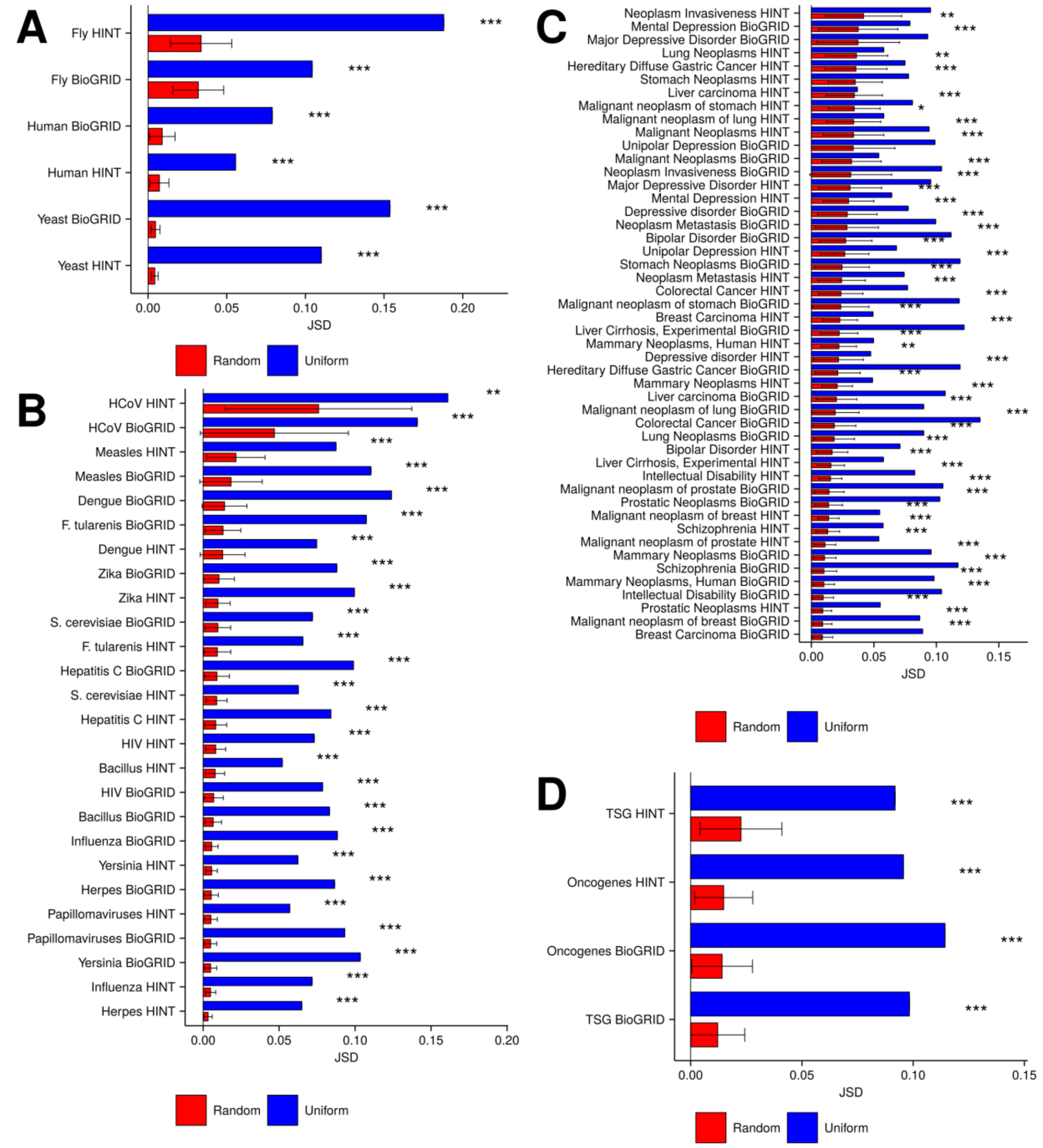

Supplementary Figure S34: Comparison between *K* distribution for functionally important genes and *RanDP* null model: Jensen Shannon Divergence (JSD) between *K* distributions for functionally important genes–A) essential, B) pathogen-related, C) disease-related and D) oncogenes and TSG–and *RanDP* null model sampled genes (red). As a control, we also show JSD between functionally important genes and uniform distribution (blue). JSD is computed by first binning *K* in the species multiplex such that each bin has at least 100 genes. Binned *K* values are used for JSD computation. Error bars show 95% CI. Statistical significance, for $\alpha = 0.05$, is marked by stars. Statistical significance is estimated using a two-tailed z-test without correction for multiple hypothesis testing.

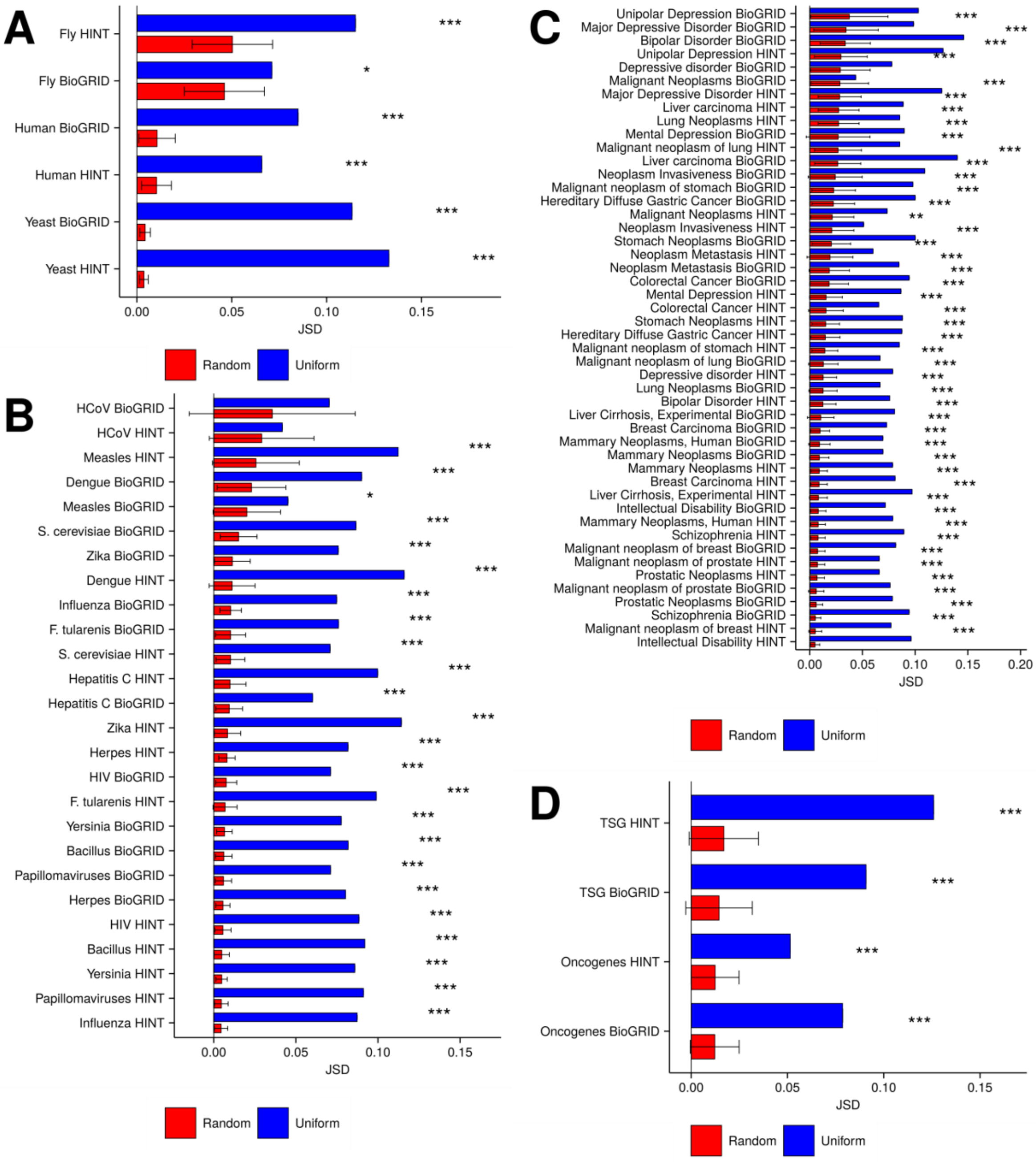

Supplementary Figure S35: Comparison between k_{in} distribution for functionally important genes and *RanDP-RZ* null model: Jensen Shannon Divergence (JSD) between k_{in} distributions for functionally important genes–A) essential, B) pathogen-related, C) disease-related and D) oncogenes and TSG–and *RanDP-RZ* null model sampled genes (red). As a control, we also show JSD between functionally important genes and uniform distribution (blue). JSD is computed by first binning k_{in} in the species multiplex such that each bin has at least 100 genes. Binned k_{in} values are used for JSD computation. Error bars show 95% CI. Statistical significance, for $\alpha = 0.05$, is marked by stars. Statistical significance is estimated using a two-tailed z-test without correction for multiple hypothesis testing.

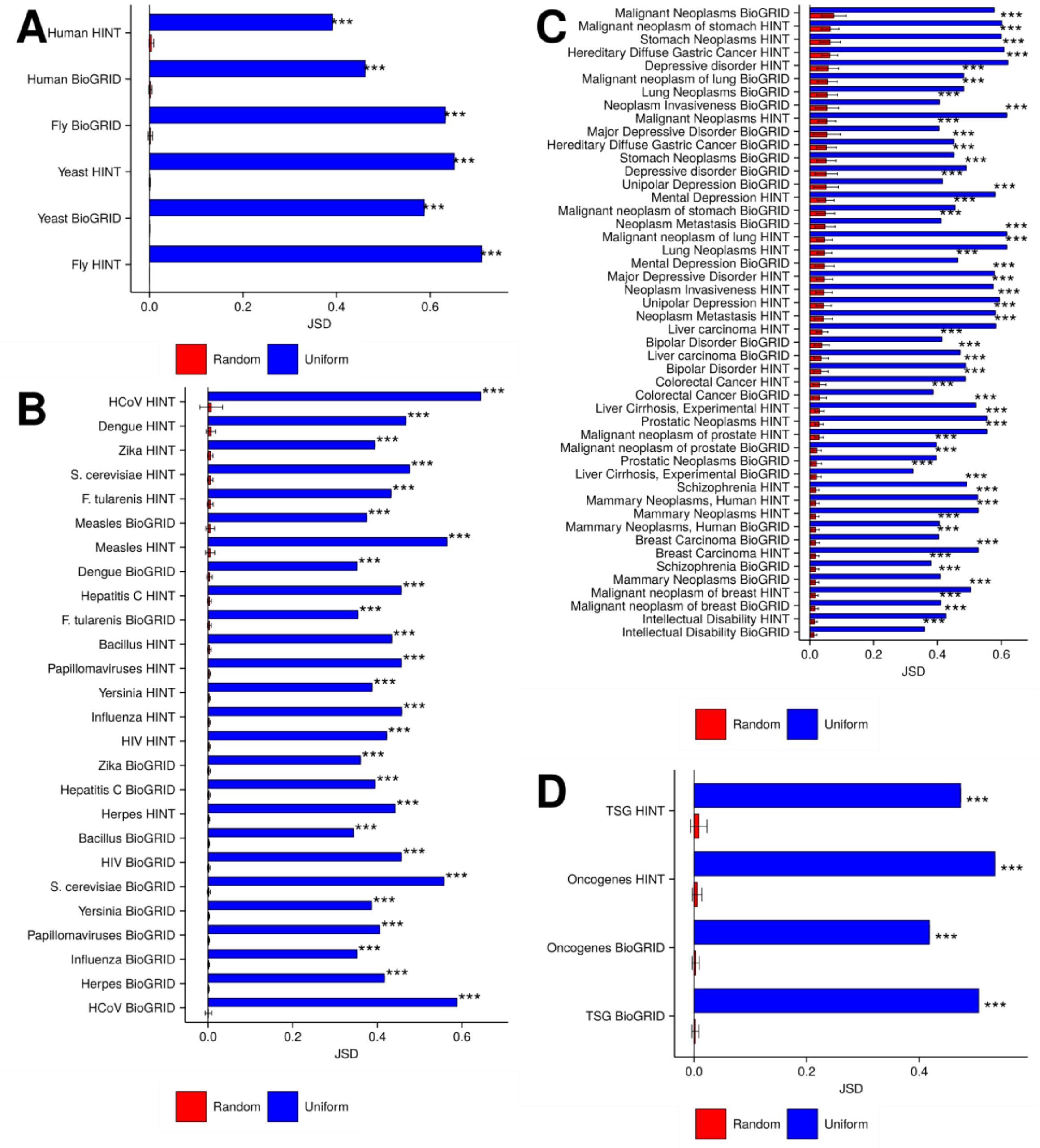

Supplementary Figure S36: Comparison between k_{out} distribution for functionally important genes and *RanDP-RZ* null model: Jensen Shannon Divergence (JSD) between k_{out} distributions for functionally important genes–A) essential, B) pathogen-related, C) disease-related and D) oncogenes and TSG–and *RanDP-RZ* null model sampled genes (red). As a control, we also show JSD between functionally important genes and uniform distribution (blue). JSD is computed by first binning k_{out} in the species multiplex such that each bin has at least 30 genes. Binned k_{out} values are used for JSD computation. Error bars show 95% CI. Statistical significance, for $\alpha = 0.05$, is marked by stars. Statistical significance is estimated using a two-tailed z-test without correction for multiple hypothesis testing.

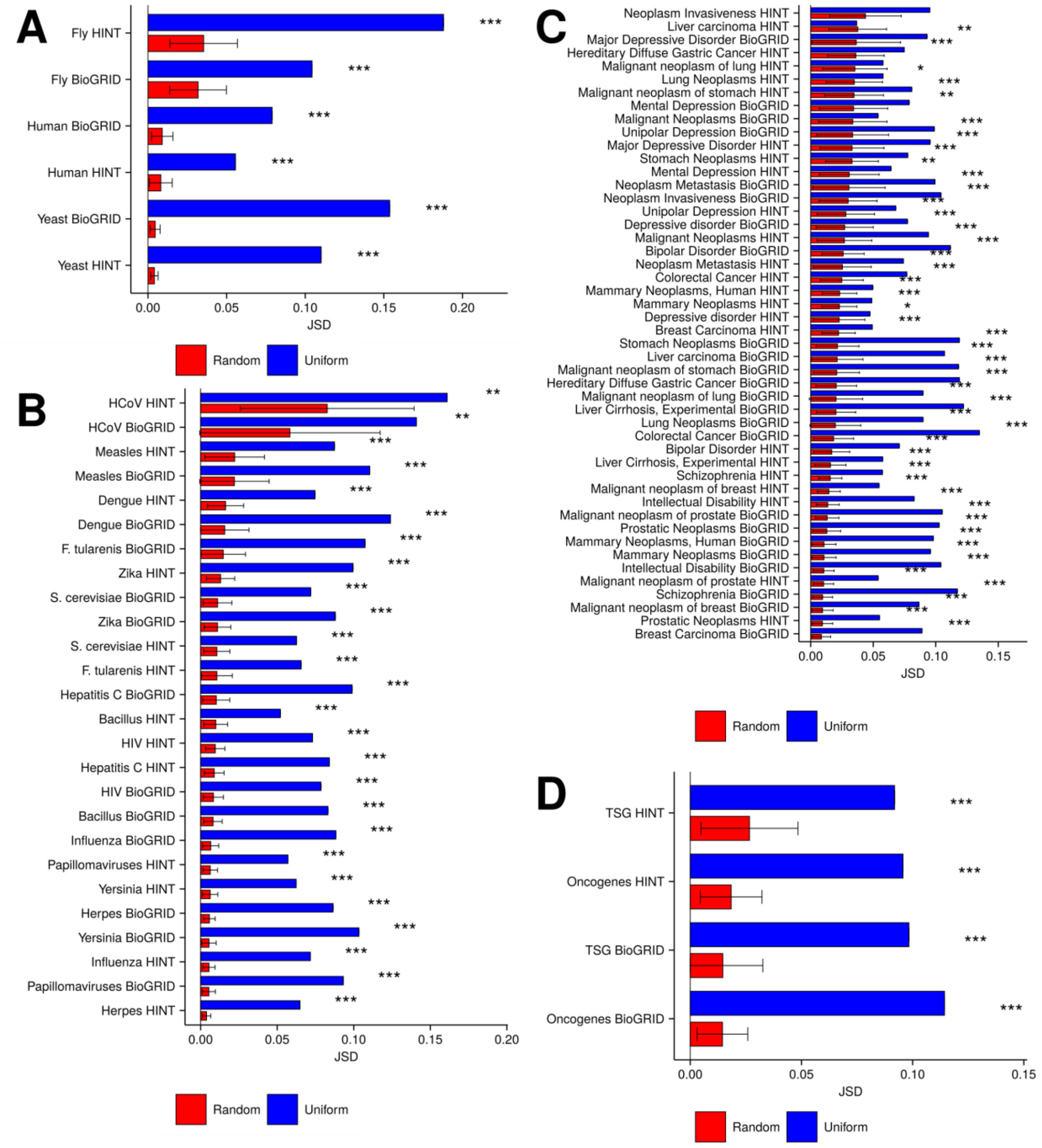

Supplementary Figure S37: Comparison between *K* distribution for functionally important genes and *RanDP-RZ* null model: Jensen Shannon Divergence (JSD) between *K* distributions for functionally important genes–A) essential, B) pathogen-related, C) disease-related and D) oncogenes and TSG–and *RanDP-RZ* null model sampled genes (red). As a control, we also show JSD between functionally important genes and uniform distribution (blue). JSD is computed by first binning *K* in the species multiplex such that each bin has at least 100 genes. Binned *K* values are used for JSD computation. Error bars show 95% CI. Statistical significance, for α = 0.05, is marked by stars. Statistical significance is estimated using a two-tailed z-test without correction for multiple hypothesis testing.

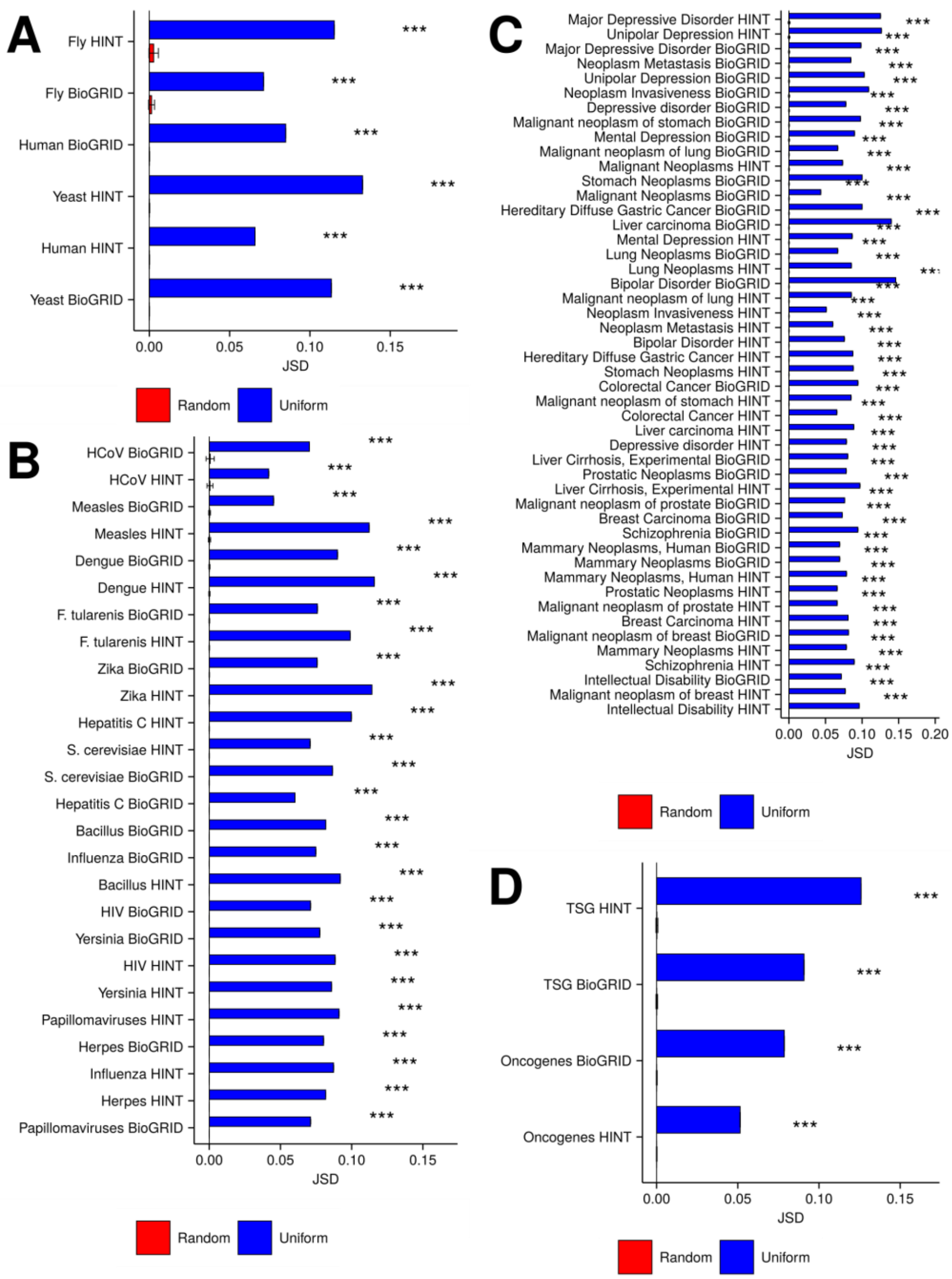

Supplementary Figure S38: Comparison between k_{in} distribution for functionally important genes and *RanDP-noC$_D$* null model: Jensen Shannon Divergence (JSD) between k_{in} distributions for functionally important genes–A) essential, B) pathogen-related, C) disease-related and D) oncogenes and TSG–and *RanDP-noC$_D$* null model sampled genes (red). As a control, we also show JSD between functionally important genes and uniform distribution (blue). JSD is computed by first binning k_{in} in the species multiplex such that each bin has at least 100 genes. Binned k_{in} values are used for JSD computation. Error bars show 95% CI. Statistical significance, for $\alpha = 0.05$, is marked by stars. Statistical significance is estimated using a two-tailed z-test without correction for multiple hypothesis testing.

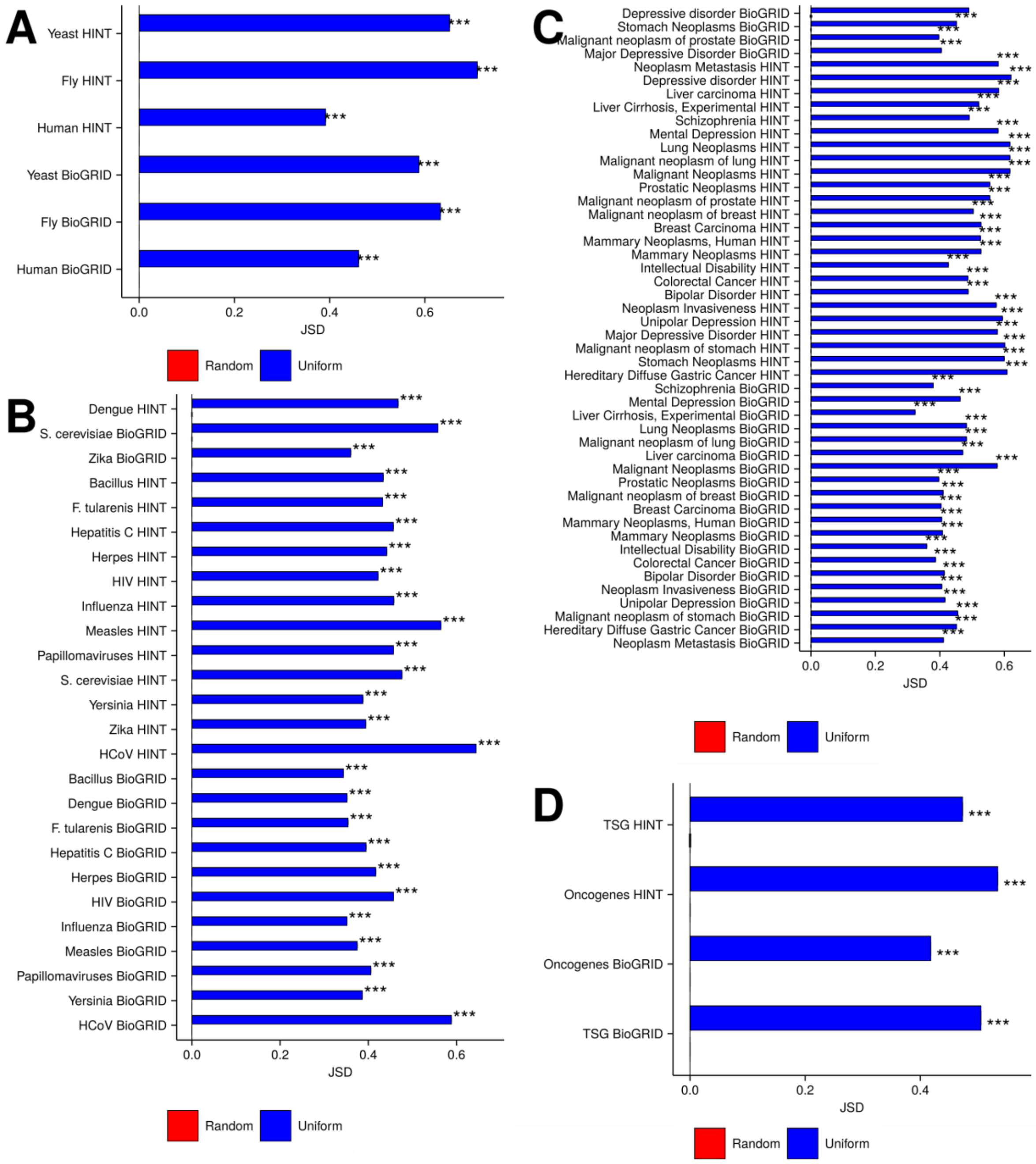

Supplementary Figure S39: Comparison between k_{out} distribution for functionally important genes and *RanDP-noC* null model: Jensen Shannon Divergence (JSD) between k_{out} distributions for functionally important genes–A) essential, B) pathogen-related, C) disease-related and D) oncogenes and TSG–and *RanDP-noC* null model sampled genes (red). As a control, we also show JSD between functionally important genes and uniform distribution (blue). JSD is computed by first binning k_{out} in the species multiplex such that each bin has at least 30 genes. Binned k_{out} values are used for JSD computation. Error bars show 95% CI. Statistical significance, for $\alpha = 0.05$, is marked by stars. Statistical significance is estimated using a two-tailed z-test without correction for multiple hypothesis testing.

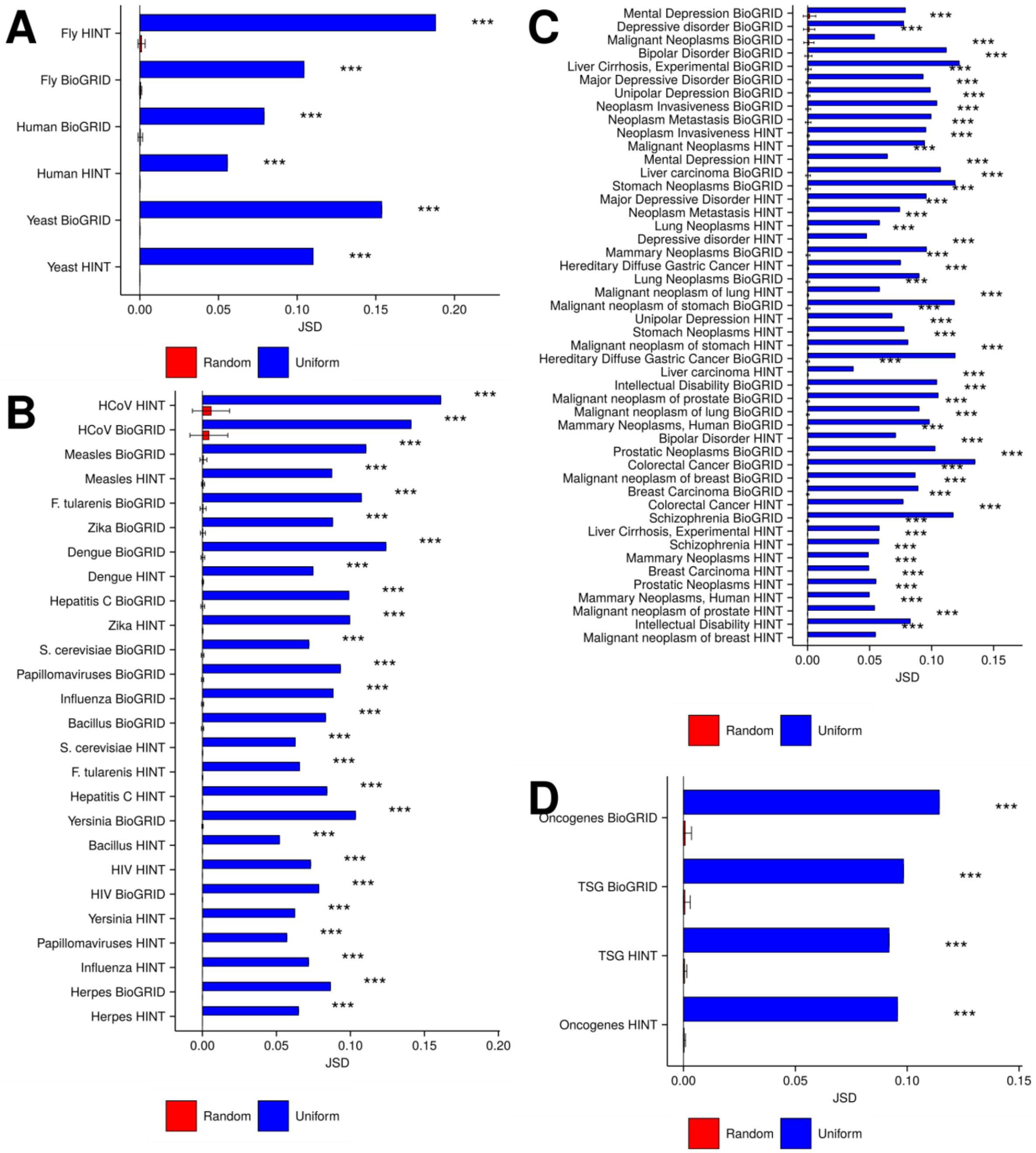

Supplementary Figure S40: Comparison between *K* distribution for functionally important genes and *RanDP-noC* null model: Jensen Shannon Divergence (JSD) between *K* distributions for functionally important genes–A) essential, B) pathogen-related, C) disease-related and D) oncogenes and TSG–and *RanDP-noC* null model sampled genes (red). As a control, we also show JSD between functionally important genes and uniform distribution (blue). JSD is computed by first binning *K* in the species multiplex such that each bin has at least 100 genes. Binned *K* values are used for JSD computation. Error bars show 95% CI. Statistical significance, for α = 0.05, is marked by stars. Statistical significance is estimated using a two-tailed z-test without correction for multiple hypothesis testing.

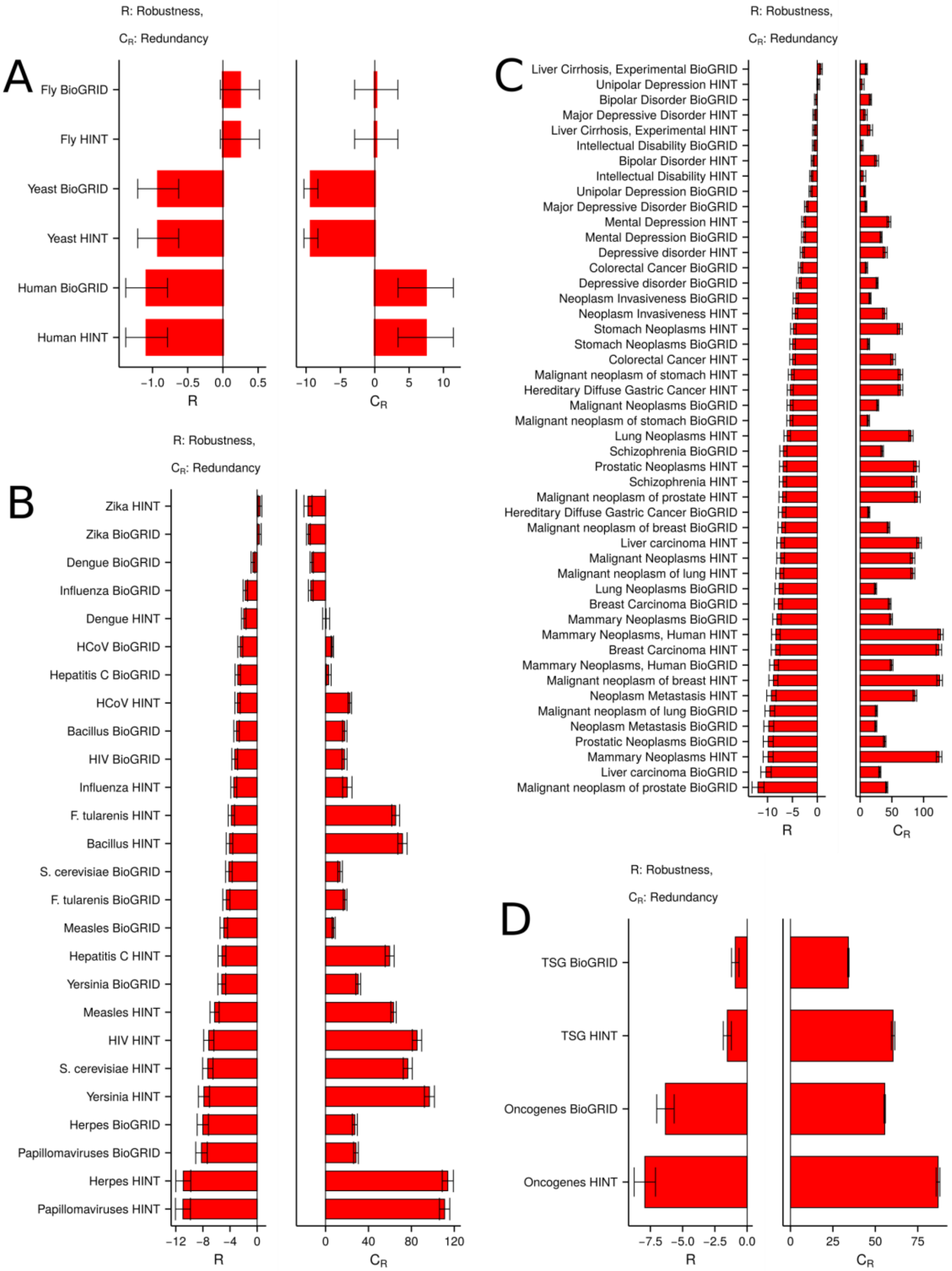

Supplementary Figure S41: Functionally important genes and proteins are redundant and essential against *RanDP-noC$_D$* null model. A) (Left) Yeast and human Essential genes and proteins have lower robustness (R) to targeted attack against *RanDP-noC$_D$* null model. (Right) Lower R is accompanied by higher redundancy (C_R) for human essential genes and proteins against *RanDP-noC$_D$* null model. Whereas for yeast, lower R is accompanied by lower redundancy (C_R). Panels B-D show results for the human multiplex. B) (Left) Pathogen-related genes and proteins

have lower R to targeted attack for most pathogens against *RanDP-noC$_D$* null model. (Right) Lower R is accompanied by higher C_R for pathogen genes and proteins against *RanDP-noC$_D$* null model. C) (Left) Disease-related genes and proteins have lower R to targeted attack for most diseases against *RanDP-noC$_D$* null model. (Right) Lower R is accompanied by higher C_R for disease genes and proteins against *RanDP-noC$_D$* null model. D) (Left) Oncogenes (tumor suppressor genes (TSGs)) and proteins have lower R to targeted attack against *RanDP-noC$_D$* null model. (Right) Lower R is accompanied by higher C_R for oncogenes (TSGs) and proteins against *RanDP-noC$_D$* null model. In all the panels, database used for PPI networks is annotated on the y-axis. In all the panels, relative robustness (R) is measured against *RanDP-noC$_D$* null model, and C_R values are calculated as the difference in redundancy against the null model. Error bars show 95% CIs. For all the panels, C_R is calculated as the mean difference in the number of redundant edges between the functionally important gene-protein pairs and the null model.

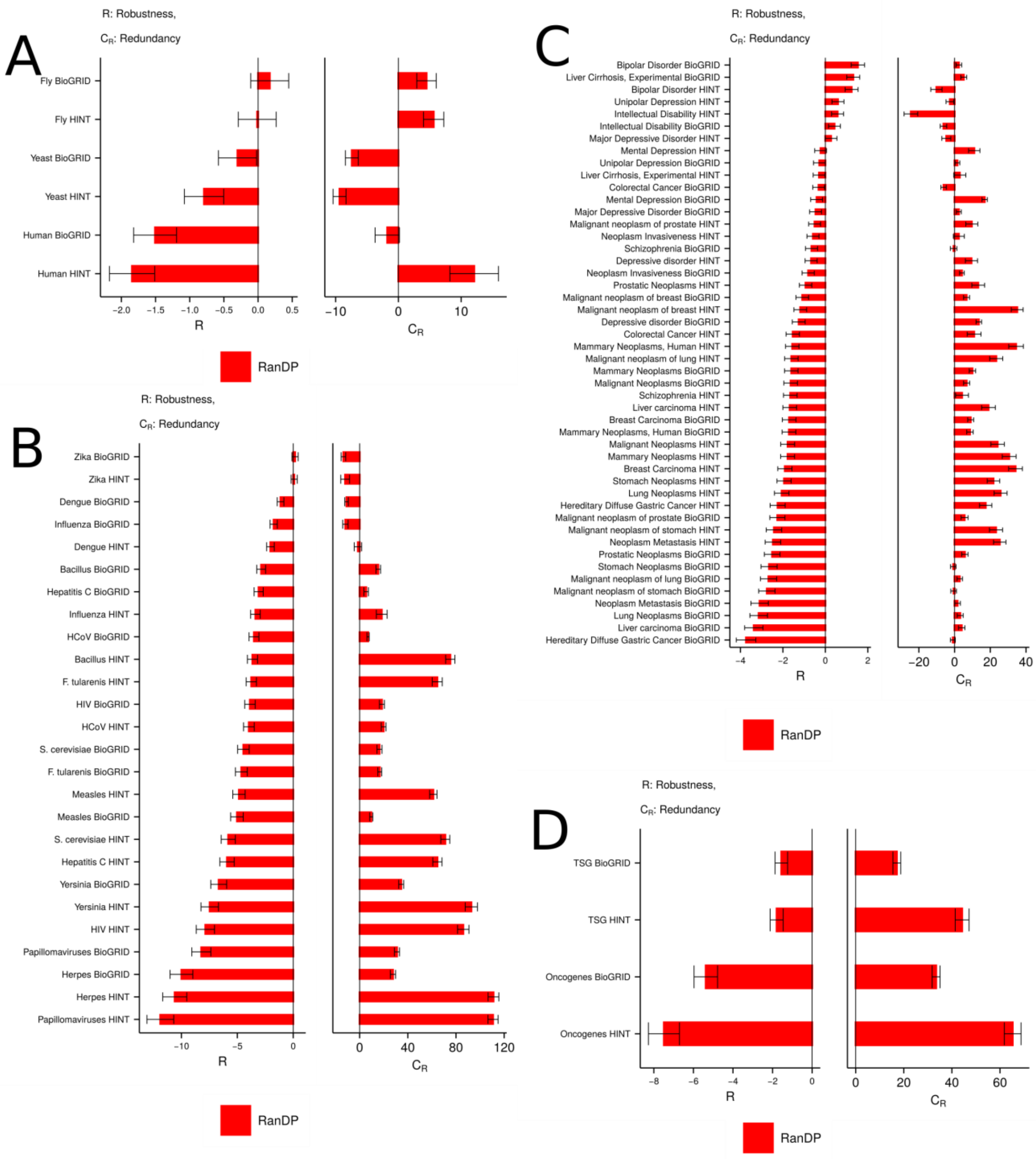

Supplementary Figure S42: Functionally important genes and proteins are redundant and essential against *RanDP* null model. A) (Left) Yeast and human Essential genes and proteins have lower robustness (R) to targeted attack against *RanDP* null model. (Right) Lower R is accompanied by higher redundancy (C_R) for human essential genes and proteins against *RanDP* null model. Whereas for yeast, lower R is accompanied by lower redundancy (C_R). Panels B-D show results for human multiplex. B) (Left) Pathogen-related genes and proteins have lower R to targeted attack for most pathogens against *RanDP* null model. (Right) Lower R is accompanied

by higher C_R for pathogen genes and proteins against *RanDP* null model. C) (Left) Disease-related genes and proteins have lower R to targeted attack for most diseases against *RanDP* null model. (Right) Lower R is accompanied by higher C_R for disease genes and proteins against *RanDP* null model. D) (Left) Oncogenes (tumor suppressor genes (TSGs)) and proteins have lower R to targeted attack against *RanDP* null model. (Right) Lower R is accompanied by higher C_R for oncogenes (TSGs) and proteins against *RanDP* null model. In all the panels, database used for PPI networks is annotated on the y-axis. In all the panels, relative robustness (R) is measured against *RanDP* null model, and C_R values are calculated as the difference in redundancy against the null model. Error bars show 95% CIs. For all the panels, C_R is calculated as the mean difference in the number of redundant edges between the functionally important gene-protein pairs and the null model.

References

1. Danielli A, Amore G, Scarlato V. Built shallow to maintain homeostasis and persistent infection: insight into the transcriptional regulatory network of the gastric human pathogen Helicobacter pylori. PLoS pathogens. 2010;6:e1000938.

2. Häuser R, Ceol A, Rajagopala SV, Mosca R, Siszler G, Wermke N, et al. A second-generation protein–protein interaction network of helicobacter pylori. Molecular & cellular proteomics. 2014;13:1318–1329.

3. Sanz J, Navarro J, Arbués A, Martín C, Marijuán PC, Moreno Y. The transcriptional regulatory network of Mycobacterium tuberculosis. PloS one. 2011;6:e22178.

4. Wang Y, Cui T, Zhang C, Yang M, Huang Y, Li W, et al. Global protein- protein interaction network in the human pathogen Mycobacterium tuberculosis H37Rv. Journal of proteome research. 2010;9:6665–6677.

5. Salgado H, Martínez-Flores I, Bustamante VH, Alquicira-Hernández K, García-Sotelo JS, García-Alonso D, et al. Using RegulonDB, the Escherichia coli K-12 Gene Regulatory Transcriptional Network Database. Current protocols in bioinformatics. 2018;61:1–32.

6. Rajagopala SV, Sikorski P, Kumar A, Mosca R, Vlasblom J, Arnold R, et al. The binary protein-protein interaction landscape of Escherichia coli. Nature biotechnology. 2014;32:285.

7. Das J, Yu H. HINT: High-quality protein interactomes and their applications in understanding human disease. BMC systems biology. 2012;6:92.

8. Teixeira MC, Monteiro PT, Palma M, Costa C, Godinho CP, Pais P, et al. YEASTRACT: an upgraded database for the analysis of transcription regulatory networks in Saccharomyces cerevisiae. Nucleic acids research. 2017;46:D348–D353.

9. Chatr-Aryamontri A, Oughtred R, Boucher L, Rust J, Chang C, Kolas NK, et al. The BioGRID interaction database: 2017 update. Nucleic acids research. 2017;45:D369–D379.

10. Bass JIF, Pons C, Kozlowski L, Reece-Hoyes JS, Shrestha S, Holdorf AD, et al. A gene-centered C. elegans protein–DNA interaction network provides a framework for functional predictions. Molecular systems biology. 2016;12.

11. Murali T, Pacifico S, Yu J, Guest S, Roberts GG, Finley RL. DroID 2011: a comprehensive, integrated resource for protein, transcription factor, RNA and gene interactions for Drosophila. Nucleic acids research. 2010;39:D736–D743.

12. Jin J, He K, Tang X, Li Z, Lv L, Zhao Y, et al. An Arabidopsis transcriptional regulatory map reveals distinct functional and evolutionary features of novel transcription factors. Molecular biology and evolution. 2015;32:1767–1773.

13. Han H, Cho J-W, Lee S, Yun A, Kim H, Bae D, et al. TRRUST v2: an expanded reference database of human and mouse transcriptional regulatory interactions. Nucleic acids research. 2017;46:D380–D386.

14. Kirkpatrick S, Gelatt CD, Vecchi MP. Optimization by simulated annealing. science. 1983;220:671–680.
